# TaPHL7 Transcription Factor Regulates Utilisation of Nitrogen and Phosphorus in Wheat

**DOI:** 10.1111/pbi.70493

**Published:** 2025-12-19

**Authors:** Huali Wang, Zhiyong Zhang, Yafei Guo, Qing Wang, Xiaochun Wang, Xiaohui Ma, Jinqiang Nian, Shuping Xiong, Xinbo Lin, Yingyin Yao, Zhongfu Ni, Fei Lu, Jianru Zuo, Xinming Ma

**Affiliations:** ^1^ State Key Laboratory of High‐Efficiency Production of Wheat‐Maize Double Cropping, Collaborative Innovation Center of Henan Grain Crops, College of Agronomy Henan Agricultural University Zhengzhou China; ^2^ State Key Laboratory of Seed Innovation, Institute of Genetics and Developmental Biology Chinese Academy of Sciences Beijing China; ^3^ Laboratory of Advanced Breeding Technologies, Institute of Genetics and Developmental Biology Chinese Academy of Sciences Beijing China; ^4^ Frontiers Science Center for Molecular Design Breeding, Key Laboratory of Crop Heterosis and Utilization (MOE), Key Laboratory of Crop Genetic Improvement China Agricultural University Beijing China; ^5^ College of Advanced Agricultural Sciences University of Chinese Academy of Sciences Beijing China

**Keywords:** artificial selection, grain yield, nitrogen, phosphorus, TaGS1;3, TaPHL7, wheat

## Abstract

Macronutrients nitrogen and phosphorus are essential for plant growth and development, thus being crucial for the productivity of crops. However, the molecular mechanism regulating the utilisation of nitrogen and phosphorus remains elusive. Here, we show that the wheat ( *Triticum aestivum* L.) transcription factor PHOSPHATE STARVATION RESPONSE‐LIKE7 (TaPHL7) regulates nitrogen and phosphorus utilisation. TaPHL7 binds to the promoter of *TaGS1;3*, encoding a key enzyme of nitrogen assimilation, to repress its expression. Nitrate relieves the transcriptional repression on a subset of nitrogen utilisation genes imposed by TaPHL7. Conversely, TaPHL7 activates the expression of a subset of Pi transporter genes, thus promoting both nitrogen utilisation and Pi acquisition. In developing seeds, *TaPHL7* expression is progressively attenuated, leading to the increased expression of *TaGS1;3*, thus enhancing nitrogen reassimilation. Notably, mutations in *TaPHL7* cause increased nitrogen remobilisation efficiency, early maturation and accelerated grain filling, eventually boosting grain yield. Moreover, *TaPHL7‐1A* has been subjected to artificial selection during wheat breeding. We propose that TaPHL7 regulates the utilisation of nitrogen and phosphorus, thus representing a promising target for genetic improvement of wheat.

## 1 Introduction

In plants, the most influential macronutrients for growth and development are nitrogen, phosphorus and potassium, all of which are absorbed by plants from soil. These macronutrients, essential elements for all living organisms, are key limiting factors for plant growth and ultimately for crop production, including wheat ( *Triticum aestivum* L.), the most widely cultivated crop providing stable food worldwide. The utilisation of these macronutrients is implemented and regulated by distinctive systems and mechanisms.

The utilisation of nitrogen in plants consists of multiple tightly interconnected processes, involving uptake, translocation, assimilation and remobilisation (Li et al. 2025; Xu et al. 2012). Upon uptake into plant cells from soil, inorganic nitrogen is incorporated into organic compounds catalysed by glutamine synthetase (GS), a central player of the GS/glutamine‐2‐oxoglutarate aminotransferase (GOGAT) cycle dominating cellular ammonium assimilation. In higher plants, two isoforms of GSs play non‐redundant functions. The plastidic GS2, usually encoded by a single‐copied nuclear gene, predominately functions in assimilating ammonium released from nitrate reduction or photorespiration in leaves. The cytosolic GS1s, encoded by multiple genes, differentiate specialised functions in response to different developmental cues (Krapp 2015; Liu et al. 2022). In cereal crops, the GS1 isoenzyme plays a key function in reassimilating ammonium released from protein degradation during leaf senescence, making a large contribution of nitrogen sources for grain development. Although genetic manipulations of the *GS1* genes have led to various alterations in metabolism or phenotypes in different species (Liu et al. 2022), the specific physiological role of each isoenzyme remains to be elucidated. In rice, a transcriptional factor OsGRF4 directly activates the expression of *OsGS1;2* and also photosynthetic genes to integrate nitrogen and carbon assimilation (Li et al. 2018). The transcriptional factor Nhd1 represses the *OsFd‐GOGAT* expression to balance nitrogen utilisation and nitrogen‐modulated flowering (Zhang, Zhang, Li, Yan, et al. 2021). In wheat, an NAC transcriptional factor TaNAC2‐5A positively regulates the *TaGS2* expression to enhance grain yield (He et al. 2015). Although genetic manipulations and transcriptional regulations of *GS* and *GOGAT* in controlling nitrogen assimilation have been extensively studied, precise regulation of spatiotemporal expression patterns of *GS* remains to be elucidated.

Phosphorus (Pi), another essential macronutrient of plants, is the most limiting nutrient for growth and development due to low mobility and availability in soil. To cope with Pi deficiency stress, plants have evolved complicated local and systematic signalling mechanisms to regulate the acquisition of inorganic Pi and the maintenance of cellular Pi homeostasis (Guo et al. 2025; Jia et al. 2023; Zeng et al. 2025). Of those key components, PHOSPHATE STARVATION RESPONSE proteins (PHRs), a class of MYB coiled‐coil (MYB‐CC) transcription factors, are characterised as master regulators of systemic Pi signalling in land plants and algae (Guo et al. 2025; Jia et al. 2023; Rubio et al. 2001). Under Pi‐sufficient conditions, the synthesis of inositol polyphosphates (InsPs) activates the interaction between the InsP sensor SPX‐domain‐containing proteins (SPXs) and PHRs (Wild et al. 2016), either restricting PHRs in the cytoplasm or repressing PHRs binding to the downstream Pi‐starvation‐response (PSR) genes in the nucleus, thereby deactivating the transcription of the PSR genes (Wang, Chen, and Wu 2021). Under Pi‐deficient conditions, PHRs are released via ubiquitin‐mediated proteasomal degradation of SPXs and thus activate expression of the PSR genes, accompanying the activation of the phosphate transporters (PHTs). In rice, a protein kinase CK2 phosphorylates PHT1 transporter and inhibits its interaction with a traffic facilitator PHF1, thus restricting PHT1 in the endoplasmic reticulum under Pi‐sufficient conditions, while Pi deficiency promotes CK2 degradation and PHT1 trafficking to the plasma membrane (Chen et al. 2015). AtVPT1 and OsVPE1/2 are shown as vacuolar Pi influx and efflux transporters, respectively, modulating Pi signalling and homeostasis under varying Pi conditions (Xu et al. 2019). Moreover, a rice phosphorus distribution transporter SPDT localised in nodes functions as a switch to control Pi allocation from leaves to grains (Yamaji et al. 2017), and a clade of protein kinase IPCKs governs InsPs biogenesis and accumulation in seeds (Xu et al. 2024). These fine‐tuned regulatory mechanisms ensure plants adapt to fluctuant Pi environment for optimised growth.

Physiologically, nitrogen nutrition and phosphorus nutrition are tightly linked and reciprocally regulated. Pi starvation generally disturbs nitrogen uptake and translocation in crops (de Magalhães et al. 1998; Rufty et al. 1990, 1991, 1993; Zeng et al. 2025), implying an intricate molecular regulatory network underlying homeostasis of these nutrients. In *Arabidopsis*, an E3 ubiquitin ligase NITROGEN LIMITATION ADAPTATION (NLA) mediates the degradation of the PHT1 transporter to regulate cellular phosphorus homeostasis in a nitrate‐dependent manner (Kant et al. 2011; Lin et al. 2013; Peng et al. 2007). Moreover, a GARP‐type transcription factor NITRATE‐INDUCIBLE GARP‐TYPE TRANSCRIPTIONAL REPRESSOR1 (NIGT1) positively regulates the expression of *PHT1* but represses the expression of *NRT2* during Pi starvation, leading to increased uptake of Pi but decreased uptake of nitrate (Wang et al. 2020). Interestingly, NIGT1 is in turn regulated by both NLP7 and PHR1, two master regulators in nitrate and Pi signalling, thereby integrating nitrogen and phosphorus signalling to regulate nitrate and Pi uptake (Maeda et al. 2018; Medici et al. 2015). In rice, Pi deficiency promotes the ubiquitination and degradation of OsSPX4, thereby releasing OsPHR2 into the nucleus and activating the expression of PSR genes (Lv et al. 2014). Notably, nitrate enhances interactions between OsSPX4 and the nitrate sensor OsNRT1.1B, recruiting an OsNRT1.1B‐interacting E3 ligase to ubiquitinate OsSPX4 and subsequent degradation, which also releases OsPHR2 into the nucleus to initiate Pi signalling (Hu et al. 2019). A similar mechanism is also operated in wheat, in which the TaTCP6 transcription factor regulates the PSR genes and nitrate‐responsive genes by releasing TaPHR2 from a TaSPX1/4‐containing complex, thus promoting utilisation of nitrate and phosphorus and eventually grain yield (Liu et al. 2025). These studies suggest that OsNRT1.1B and OsSPX4 play important roles in coordinating nitrogen and phosphorus signalling. While these efforts highlight the importance of the nitrogen–phosphorus interaction during the nutrient acquisition processes, little is known about other physiological processes such as assimilation and remobilisation.

In this study, we find TaPHL7, a PHR1‐like protein, acting as a negative transcriptional regulator of *TaGS1;3* in wheat. TaPHL7 plays a dual role by positively regulating PSRs and negatively regulating nitrogen uptake, translocation and assimilation. We find that nitrate relieves the inhibitory role of TaPHL7 on nitrogen utilisation genes and promotes the expression of a subset of *PHT* genes. Consequently, mutations in *TaPHL7* cause a higher nitrogen remobilisation efficiency (NRE) in developing grains and eventually boost grain yield. Remarkably, *TaPHL7‐1A* underwent artificial selection during wheat breeding. These results suggest that TaPHL7 plays an important role in modulating the balance of nitrogen and phosphorus nutrition, thus representing a promising target for molecular breeding of elite wheat varieties.

## 2 Results

### 2.1 TaPHL7 Represses *TaGS1;3* Transcription

**FIGURE 1:**
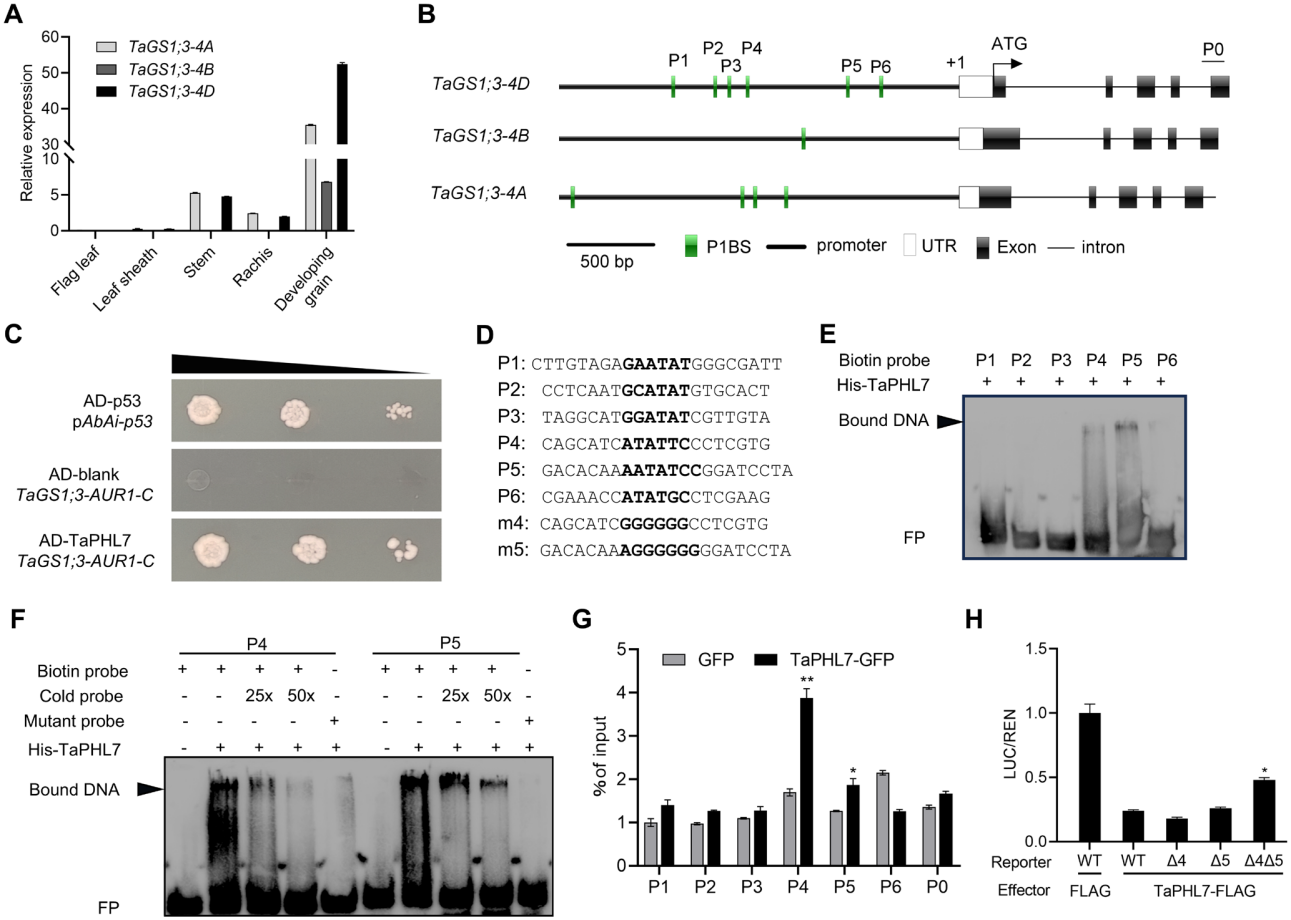
TaPHL7 represses *TaGS1;3* transcription. (A) Relative expression levels of three *TaGS1;3* homologous genes in the indicated organs and tissues at 16‐day post anthesis analysed by RT‐qPCR. Data are mean values with s.d. obtained from three technical replicates and each replicate consists of at least six individuals. (B) Structural schemes of three *TaGS1;3* homologous genes. The putative P1BS elements in the promoter are indicated by green bars. Open and filled boxes indicate the 5′‐untranslated and coding regions, respectively. (C) Growth of yeast cells co‐transformed with the indicated vectors. The transformed cells are 10‐, 100‐ and 1000‐fold diluted (from left to right), respectively, and then grown on SD/−Leu medium containing 200 ng/mL of aureobasidin A. (D) DNA sequences of probes containing wild‐type (P1 to P6) and mutated (m4 and m5) P1BS elements used in EMSA assay. The putative wild‐type and mutated P1BS elements are shown in bold. (E, F) EMSA with recombinant His‐TaPHL7 protein and DNA probes showing in (D). Arrowheads indicate protein‐DNA complexes. FP, free probe. (G) Analysis of the binding activity of TaPHL7‐GFP protein to the indicated P1BS elements or DNA fragments by ChIP‐qPCR. DNA fragments were immunoprecipitated using an anti‐GFP antibody and then subjected to qPCR analysis using fragments‐specific primers. Data are means of two biological replicates with s.d. (H) Repression of the *TaGS1;3* expression by TaPHL7‐FLAG. Tobacco leaves were transiently transformed with the *35S::TaPHL7‐FLAG* (TaPHL7‐FLAG) expression vector or an empty vector (FLAG) along with a reporter plasmid of *proTaGS1;3::LUC* (WT) or mutants (Δ4, Δ5 and Δ4Δ5) containing a *REN* reference gene. The relative LUC activity of Control was set as 1.0. Data are means of three biological replicates with s.d. * and ** indicate *p* < 0.05 and *p* < 0.01 (Student's *t*‐test), respectively.

In a previous study, we found that the transcription of three *TaGS1* genes, *TaGS1;1*, *TaGS1;2* and *TaGS1;3*, showed variable levels at different stages in developing grains. Moreover, the spatial distributions of the isoenzymes encoded by these three genes also showed distinctive patterns, suggestive of their functional divergences (Wei et al. 2021). In a transcriptome analysis, we found that *TaGS1;1* (except *TaGS1;1‐6D*) and *TaGS1;2* exhibited relatively high expression levels in vegetative organs/tissues, but with significantly reduced expression levels in developing grains (Figure S1A). By contrast, *TaGS1;3* showed a relatively lower expression level in vegetative organs/tissues but was specifically expressed in developing grains (Figure S1A). An RT‐qPCR analysis further showed that all three homoalleles of *TaGS1;3* genes (*TaGS1;3–4A*, *TaGS1;3–4B* and *TaGS1;3–4D*; *TraesCS4A02G266900*, *TraesCS4B02G047400* and *TraesCS4D02G047400*) were preferentially expressed in developing grains (Figure 1A,B), suggesting that *TaGS1;3* may play an important role in regulating seed development.

To identify transcription factors regulating the expression of *TaGS1;3*, we performed a yeast one‐hybrid (Y1H) screen using the *TaGS1;3–4D* promoter (with the highest expression level in developing grains; see Figure 1A) as a bait sequence (2000 bp upstream from the putative transcription start; Figure 1B). Among the identified candidates (Table S1), a MYB‐CC‐type transcription factor PHR1‐like7 (TaPHL7) was of particular interest, as its orthologs in *Arabidopsis* and rice have been shown to regulate Pi signalling (Liu et al. 2010; Rubio et al. 2001; Zhou et al. 2008). When transiently expressed in wheat protoplasts, a TaPHL7‐GFP fusion protein was localised in the nucleus (Figure S1B). When recapitulating the Y1H assay, a GAL4 transcription activation domain (AD)‐TaPHL7 fusion protein effectively activated the expression of the *TaGS1;3p*::*AUR1‐C* reporter (Figure 1C). Inspection of the *TaGS1;3–4D* promoter sequence revealed the presence of multiple imperfectly palindromic PHR1‐binding sequences (P1BS; 5′‐GNATATNC‐3′; P1 through P6) (Rubio et al. 2001) (Figure 1B,D; Figure S1C). To examine whether TaPHL7 was capable of binding to these putative P1BS elements, we performed an electrophoretic mobility shift assay (EMSA) using the matched sequences in the *TaGS1;3–4D* promoter (P1 through P6) as probes (Figure 1E). We found that HIS‐TaPHL7 recombinant protein bound to two biotin‐labelled oligo‐nucleotide probes derived from P4 and P5 sites with varying affinities, and the binding activity was substantially inhibited by cold (biotin‐free) probes (Figure 1F). Moreover, mutations of either P4 or P5 element substantially reduced the binding activity. No binding activity of HIS‐TaPHL7 was detectable in the remaining 4 putative P1BS elements (P1 through P3 and P6; Figure 1E). These results suggested that TaPHL7 directly binds to the promoter of *TaGS1;3*.

In a chromatin immunoprecipitation (ChIP) coupled with quantitative PCR (qPCR) assay, TaPHL7‐GFP protein efficiently bound to P4 and, in a less affinity, to P5, when transiently expressed in wheat protoplasts (Figure 1G). To examine the possible transcriptional activity of TaPHL7, a reporter plasmid harbouring a luciferase reporter gene (*LUC*) driven by the *TaGS1;3–4D* promoter was co‐transfected into tobacco leaves with an effector plasmid expressing of *TaPHL7‐FLAG* or an empty plasmid (Figure S1D). We found that TaPHL7‐FLAG substantially repressed the expression of *TaGS1;3‐LUC* (Figure 1H; Figure S1D). Notably, deletion of either P4 or P5 (Δ4 or Δ5) element did not have an apparent effect on the TaPHL7‐FLAG‐repressed expression of *TaGS1;3‐LUC*, implying either element is sufficient to mediate transcriptional repression by TaPHL7‐FLAG. However, the deletion of both P4 and P5 significantly relieved the expression of *TaGS1;3‐LUC* from the TaPHL7‐FLAG‐mediated repression (Figure 1H; Figure S1D). We noticed that the expression of *TaGS1;3‐LUC* was not fully restored by simultaneous deletion of P4 and P5, implying other transcription factors may also regulate *TaGS1;3* transcription. Collectively, these results suggested that TaPHL7 directly binds to the promoter of *TaGS1;3* to repress its transcription, predominately mediated by the P4 and P5 elements.

### 2.2 *TaPHL7* Regulates Plant Growth and Development

**FIGURE 2:**
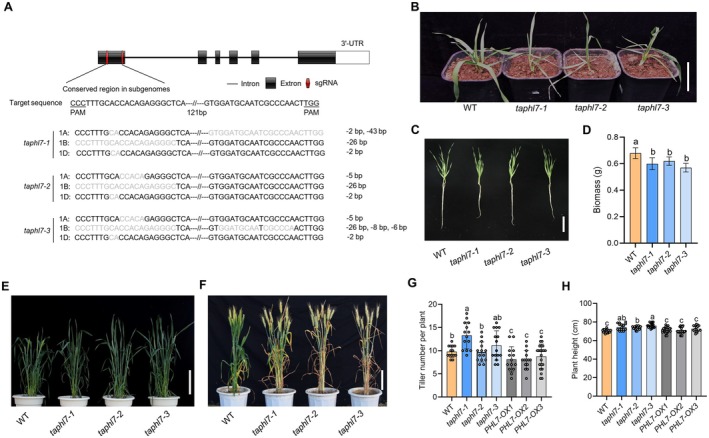
*TaPHL7* regulates plant growth and development. (A) Top: Structural scheme of the *TaPHL7* genome structure. The edited regions are shown in red in Exon 1. Bottom: DNA sequences of *taphl7‐1*, ‐*2* and ‐*3* mutants at the edited regions. Deleted regions in each allele are shown in grey, and numbers of the deleted nucleotides are shown at the right. (B) Wild‐type ZM7698 (WT) and *taphl7* mutant plants of 2‐month‐old grown in the field. Scale bar, 10 cm. (C) Wild‐type ZM7698 (WT) and *taphl7* mutant seedlings of 18‐day‐old grown under hydroponic conditions in a greenhouse. Scale bar, 10 cm. (D) Quantitative analysis of biomass of seedlings shown in (C). (E, F) Wild‐type ZM7698 (WT) and *taphl7* plants at the heading stage (E) or 28‐day post anthesis (F) were grown in natural field. Scale bars, 20 cm. (G, H) Quantitative analysis of tiller number (G) and plant height (H) of WT, *taphl7* and *TaPHL7*‐overexpressing (*PHL7‐OX*) plants at the mature stage. Data presented in (D), (G, H) are mean values with s.d. (*n* ≥ 15 plants), and different letters indicate *p* ≤ 0.05 (LSD multiple range tests).

In the wheat genome, 6 *TaPHR* genes and 11 *TaPHL* genes were identified (Figure S2A). Of those, *TaPHL7* is located on Chromosome 1 (*TraesCS1A02G309200*, *TraesCS1B02G320000* and *TraesCS1D02G308600*), referred to as *TaPHL7‐1A*, *TaPHL7‐1B* and *TaPHL7‐1D*, respectively. TaPHL7‐1A and TaPHL7‐1D share a 98.2% identity, while TaPHL7‐1B, located in a separate branch, shares 90.7% and 89.7% identities with its two homoalleles, respectively (Figure S2A,B). To assess the *TaPHL7* function, we generated *taphl7* mutations using the clustered regularly interspaced short palindromic repeats (CRISPR)/CRISPR‐associated protein Φ2 (CasΦ2) genome editing system (Zhao et al. 2024). Using two single guide RNA (sgRNA) conserved in all three wheat subgenomes, targeting Exon 1 of *TaPHL7* (Figure 2A), we generated *taphl7* mutant plants in the genetic background of Zhengmai7698 (ZM7698), a high‐yield common wheat cultivar widely cultivated in Northern China. Three transgene‐free and homozygous *taphl7* mutants were identified and characterised, designated as *taphl7‐1*, *taphl7‐2* and *taphl7‐3*, respectively, which carried frame‐shift mutations in all three subgenomes and were considered as null mutations (Figure 2A). The expression of *TaPHL7* was substantially reduced in these mutants (Figure S2C). In addition, we also generated transgenic plants overexpressing a *TaPHL7‐1A* transgene under the control of a maize *Ubiquitin* promoter in the ZM7698 background. Expression of *TaPHL7* was significantly increased in the transgenic plants (Figure S2D).

Under routine field growth conditions (240 kg/ha of urea and 750 kg/ha of compound fertiliser with nitrogen (N), phosphorus pentoxide (P_2_O_5_) and potassium oxide (K_2_O) in the compound fertiliser are ≥ 15%, 15% and 15%, respectively), all three *taphl7* mutants grew slightly smaller and showed a narrow‐leaf phenotype during the vegetative growth stage (Figure 2B; Figure S2E). When cultured under hydroponic conditions, these mutants showed a similar phenotype with reduced biomass (Figure 2C,D). At the heading stage, the *taphl7* mutants showed an increased tiller number and slightly increased plant height compared with wild‐type plants (Figure 2E–H). Although the heading date of the *taphl7* mutants was delayed for 3–5 days, the *taphl7* mutants showed an early‐maturing phenotype (Figure 2F; Figure S2F). Under the assay conditions, *TaPHL7*‐overexpressing plants did not have an apparent phenotype (Figure 2G,H; see also below). These observations suggest that *TaPHL7* plays an important role in regulating plant growth and development.

### 2.3 *TaPHL7* Positively Regulates Pi Signalling and Pi Acquisition

Since *TaPHL7* was implied as a regulator of Pi signalling, we examined the responses of *taphl7* mutants to Pi under hydroponic culture conditions with 0.20 mM KH_2_PO_4_ (normal Pi) or 0.01 mM (low Pi) (Hu et al. 2019; Lv et al. 2014; Tian et al. 2024; Wang et al. 2013).

**FIGURE 3:**
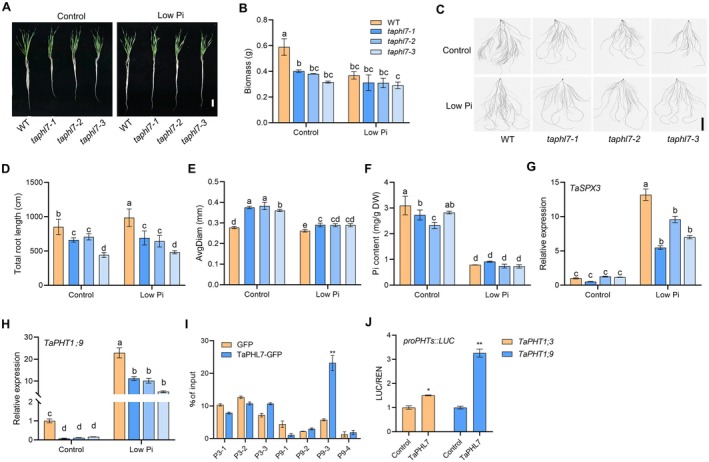
*TaPHL7* positively regulates Pi signalling and Pi acquisition. (A) Eighteen‐day‐old wild‐type ZM7698 (WT) and *taphl7* seedlings grown under Pi‐sufficient (control; 0.20 mM KH_2_PO_4_) or Pi‐deficient (low Pi; 0.01 mM KH_2_PO_4_) conditions. Seeds were imbibed for 1 day and grown in deionised water for 5 days, followed by cultured under Control or Low Pi conditions for 12 days. Scale bar, 5 cm. (B) Quantitative analysis of biomass of seedlings shown in (A). (C) Roots derived from seedlings shown in (A). Scale bar, 5 cm. (D, E) Quantitative analysis of total root length (D) and root diameter (E) of roots shown in (C). (F) Analysis of phosphorus content in roots of seedlings shown in (A). (G, H) Relative expression levels of *TaSPX3* (G) and *TaPHT1;9* (H) in roots of 18‐day‐old WT and *taphl7* mutants grown under control or low Pi condition. (I) Analysis of the binding activity of TaPHL7‐GFP protein to the *TaPHT1;3* and *TaPHT1;9* promoters by ChIP. DNA fragments were immunoprecipitated using an anti‐GFP antibody and then subjected to qPCR analysis using fragments‐specific primers as indicated. (J) Activation of the expression of *TaPHT1;3* and *TaPHT1;9* by TaPHL7‐FLAG. Tobacco leaves were co‐transfected with the p*35S::TaPHL7‐FLAG* (TaPHL7) expression vector or an empty vector (control) along with a reporter plasmid of *proTaPHT1;3::LUC* or *proTaPHT1;9::LUC* containing a *REN* reference gene, respectively. The relative LUC activity of control was set as 1.0. All data presented are means with s.d. from at least 15 seedings in (B), (D–F), three biological replicates in (G, H), (J) and two biological replicates in (I). Different letters indicate *p* ≤ 0.05 (LSD multiple range tests). *, **p < 0.05, *p* < 0.01 (Student's *t*‐test), respectively.

While wild‐type seedlings showed reduced growth under Pi‐deficient conditions, the *taphl7* mutants displayed substantially reduced responses to Pi deficiency (Figure 3A,B). Under normal (Pi‐sufficient) conditions, the *taphl7* mutants had reduced root length and increased root diameter (Figure 3C–E). Consistent with these results, total Pi contents were reduced in the roots of the *taphl7* mutants under Pi‐sufficient conditions (Figure 3F), suggesting that *TaPHL7* positively regulates Pi acquisition. When grown under Pi‐deficient conditions, while the wild type showed an increase in root length, the *taphl7* mutants marginally responded to the low Pi challenge (Figure 3C,D), suggesting that the PSR is partly dependent on *TaPHL7*. We noticed that, compared with root development, fewer apparent growth defects were observed in the aerial part of the mutants. This phenotype might result from functional compensation from other PHR‐like genes.

Data presented above suggest that *TaPHL7* may play a role in regulating Pi signalling. As the expression of *TaPHL7‐1A* and *TaPHR1* was not regulated by Pi levels (Figure S3A,B), a case similar to that previously observed in *AtPHR1* (Rubio et al. 2001), we then examined the expression of key components in the Pi signalling pathway in response to Pi starvation. The expression of the putative Pi sensor *TaSPX3* and downstream Pi transporter genes *TaPHTs* (*TaPHT1;3*, *TaPHT1;6* and *TaPHT1;9*) was highly inducible by Pi starvation (Grün et al. 2018; Liu et al. 2024; Teng et al. 2017). Similarly, the expression of these genes in wild‐type ZM7698 seedlings was dramatically induced by Pi starvation (Figure 3G,H; Figure S3C,D). While the expression of these genes showed marginal alterations in the *taphl7* mutants compared with wild type under normal Pi conditions, the Pi‐starvation‐induced expression of these genes was remarkably compromised in the *taphl7* mutants (Figure 3G,H; Figure S3C,D), suggesting that *TaPHL7* positively regulates these Pi‐responsive genes during Pi starvation.

An analysis of the promoters of *TaPHT1;3* and *TaPHT1;9*, representing high‐ or low‐responsive genes to Pi, identified several putative P1BS sites (Figure S3E). In a ChIP‐qPCR analysis, no significant binding of TaPHL7‐GFP protein was detected to the putative P1BS sites of the *TaPHT1;3* promoter, which might be attributed to yet unidentified cis‐elements or other unknown reasons. However, strong binding of TaPHL7‐GFP protein was observed in the putative P1BS site 3 of the *TaPHT1;9* promoter (Site P9‐3; Figure 3I; Figure S3E). Consistent with this observation, TaPHL7 substantially promoted the expression of the *TaPHT1;9*‐*LUC* reporter gene but slightly increased the expression of the *TaPHT1;3*‐*LUC* when transiently expressed in tobacco leaf (Figure 3J). Collectively, these results suggest that *TaPHL7* positively regulates Pi signalling and Pi acquisition.

### 2.4 *TaPHL7* Negatively Regulates Nitrogen Metabolism

**FIGURE 4:**
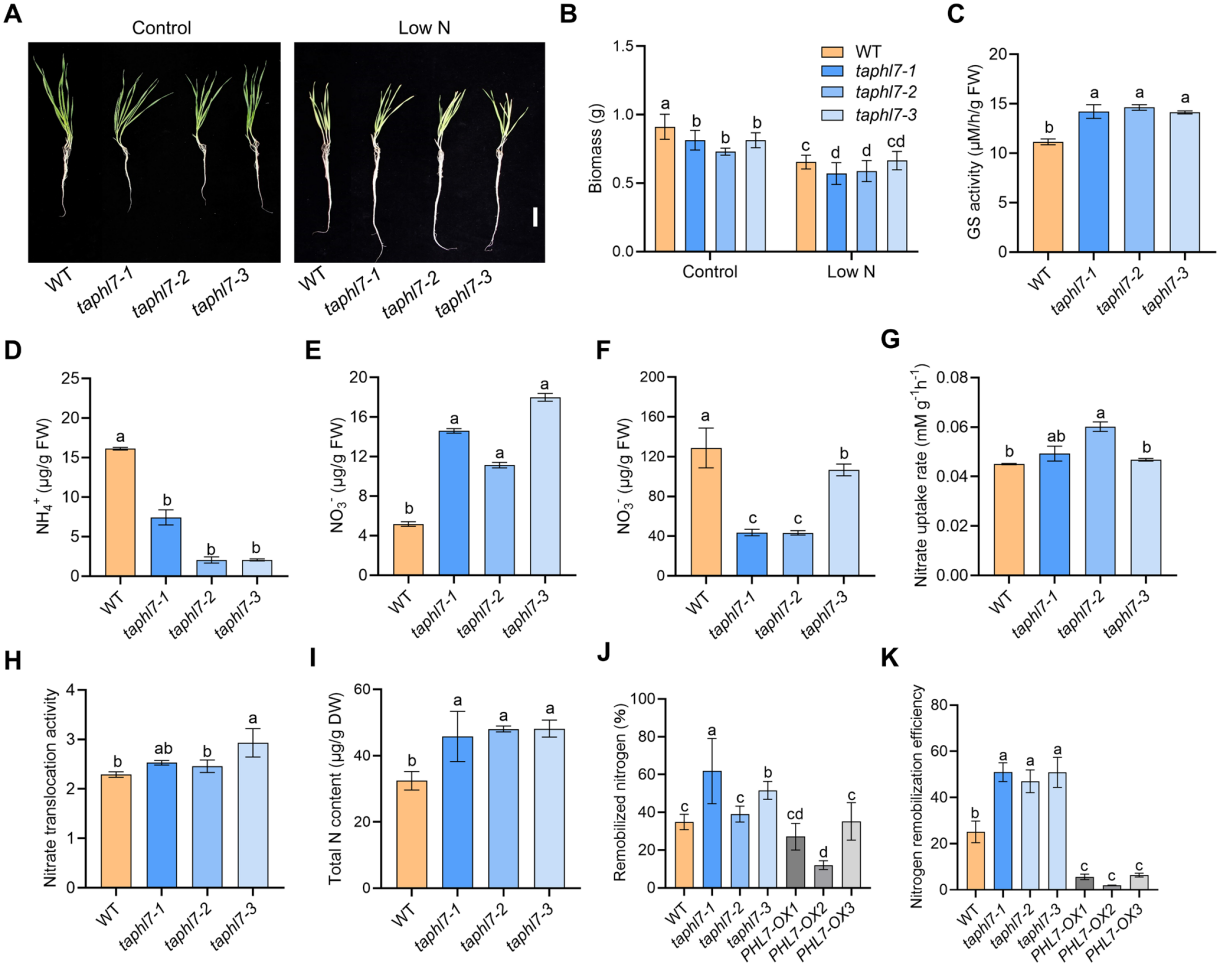
*TaPHL7* negatively regulates nitrogen metabolism. (A) Eighteen‐day‐old wild‐type ZM7698 (WT) and *taphl7* seedlings grown under N‐sufficient (control; 2.00 mM Ca(NO_3_)_2_) or N‐deficient (low N; 0.20 mM Ca(NO_3_)_2_) conditions. Scale bar, 5 cm. (B) Quantitative analysis of biomass of seedlings shown in (A). (C, D) Analysis of total GS activity (C) and the ammonium content (D) in developing grain of wild‐type ZM7698 (WT) and the *taphl7* mutants at 16 days post‐anthesis. (E, F) Analysis of the nitrate content in developing grain (E) and flag leaf (F) of WT and *taphl7* mutants grown at 16 days post‐anthesis. (G, H) Analysis of nitrate uptake rate (G) and root‐to‐shoot nitrate translocation activity (H) of 16‐day‐old WT and *taphl7* mutants. (I) Analysis of total nitrogen content in vegetative organs of WT and *taphl7* plants grown at anthesis stage. (J) Analysis of the remobilised nitrogen from vegetative organs of WT, *taphl7* and *TaPHL7*‐overexpressed (*PHL7‐OX*) plants during grain filling at 0 to 40 days post‐anthesis. (K) Analysis of nitrogen remobilisation efficiency of WT, *taphl7* and *PHL7‐OX* plants grown in field conditions. Data in (B–F) are means with s.d. (*n* = 15 seedlings in B and 5 plants in C–F). Data in (G–K) are means with s.d. obtained from three biological replicates, and each replicate consists of at least five plants. Different letters indicate *p* ≤ 0.05 (LSD multiple range tests).

Given that TaPHL7 binds to the promoter of *TaGS1;3* to repress its expression, it is reasonable to assume that TaPHL7 may also regulate nitrogen assimilation and possibly other nitrogen metabolism processes. To address this question, we examined the response of the *taphl7* mutants to nitrogen under hydroponic culture conditions. Similar to that grown under low Pi conditions, the *taphl7* mutants showed reduced responses to low N (Figure 4A,B). Because *TaGS1;3* was primarily expressed in developing grains (see Figure 1A; Figure S1A), we examined the transcription profiles of *TaPHL7* at the grain‐filling stage. In developing seeds, the expression of *TaPHL7* was decreased during the course of grain filling, in a pattern reverse to that of *TaGS1;3* (Figure S4A). Moreover, *TaPHL7* expression was also progressively decreased in all examined vegetative organs during grain filling (Figure S4A), suggesting that the reduced expression of *TaPHL7* at the post‐anthesis stage may relieve *TaGS1;3* from the transcription repression. Consistent with this notion, compared with wild type, the *taphl7* mutants had a higher level of TaGS1;3 protein and had an increased GS activity in developing grains (Figure 4C; Figure S4B,C). The ammonium content in the developing grain was significantly decreased in the *taphl7* mutants (Figure 4D), suggesting that TaPHL7 may regulate nitrogen reassimilation in developing grain by controlling the TaGS1;3 activity.

With a reduction of the ammonium level, the content of nitrate, the major inorganic nitrogen absorbed by wheat, was significantly increased in developing grains but decreased in flag leaves of the *taphl7* mutants (Figure 4E,F), suggesting that *TaPHL7* may also regulate nitrogen uptake and translocation processes, which may promote a more active flux of nitrogen from source (flag leaves) to sink (developing grain). To further test this possibility, we adopted a stable isotope tracing approach to examine the nitrogen acquisition ability at the vegetative growth stage. Compared with wild‐type seedlings, both nitrate uptake rate and root‐to‐shoot translocation activity were increased in the *taphl7* seedlings at varying degrees (Figure 4G,H), accompanied by increased expression of *TaNRT2.1* and *TaGS1;2* in *taphl7* roots (Figure S4D,E). Consequently, the total nitrogen content was increased in the *taphl7* mutants at both the seedling and flowering stages (Figure 4I; Figure S4F). In an analysis of nitrogen remobilisation, we found that the remobilised nitrogen from vegetative organs was increased in the *taphl7* mutants but decreased in *TaPHL7*‐overexpressing plants at varying degrees, associated with decreased TaGS1;3 levels in developing grains (Figure 4J; Figure S4G). Correlated to this result, the NRE was substantially increased in the *taphl7* mutants but decreased in *TaPHL7*‐overexpressing plants (Figure 4K), suggesting that a higher level of nitrogen is committed to contribute from vegetative sources to the grain sink. Taken together, these results suggest that *TaPHL7* negatively regulates nitrogen uptake, translocation and remobilisation at both vegetative growth and post‐anthesis stages.

### 2.5 *TaPHL7* ‐Regulated Nitrogen and Phosphorus Utilisation Modulates Grain Yield

**FIGURE 5:**
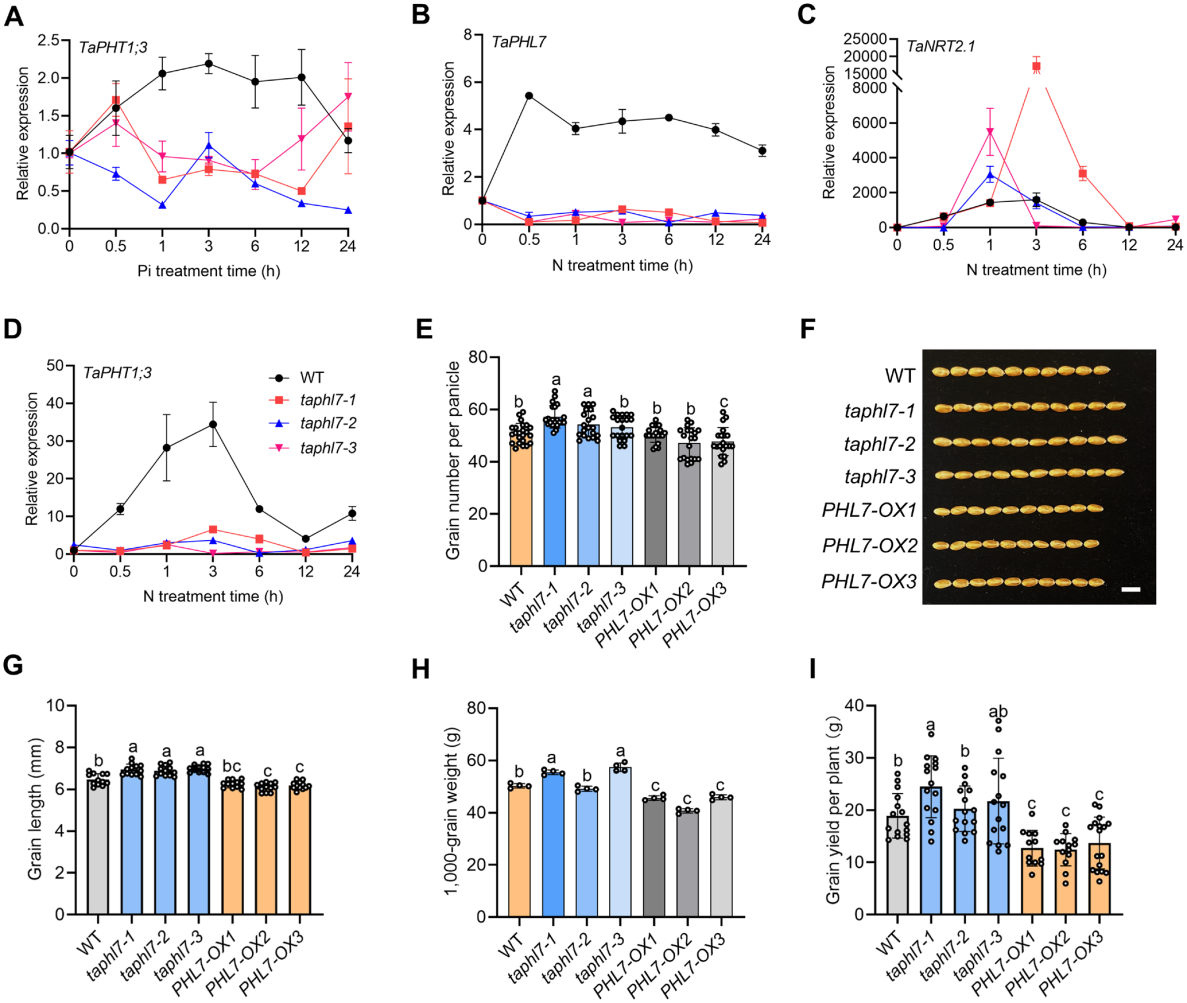
*TaPHL7* controls grain yield. (A) Expression levels of *TaPHT1;3* in roots of wild‐type ZM7698 (WT) and *taphl7* seedlings. Sixteen‐day‐old seedlings grown under phosphate‐free conditions were transferred to a solution containing 0.20 mM KH_2_PO_4_ and then cultured for the indicated times. (B–D) Expression levels of *TaPHL7* (B), *TaNRT2.1* (C) and *TaPHT1;3* (D) in roots of wild‐type ZM7698 (WT) and *taphl7* seedlings in response to nitrogen. Sixteen‐day‐old seedlings grown under nitrogen‐free solutions were transferred to a nitrogen‐containing solution (2.00 mM Ca(NO_3_)_2_) and then cultured for the indicated times. (E) Quantitative analysis of grain number per panicle of WT, *taphl7* and *PHL7‐OX* plants. (F) Mature grains with the indicated genotypes. Scale bar, 1 cm. (G, H) Statistical analysis of grain length (G) and 1000‐grain weight (H) of grains shown in (F). (I) Actual grain yield of WT, *taphl7* and *PHL7‐OX* plants in the field trial. All data presented are mean values with s.d., estimated from three technical replicates and each replicate consists of at least 3 seedlings in (A–D), and consists of at least 15 plants in (E, G–I). Different letters indicate *p* ≤ 0.05 (LSD multiple range tests).

Data presented above suggest that *TaPHL7* negatively and positively regulates nitrogen and phosphorus signalling, respectively. We observed that the loss of *TaPHL7* function caused a greater sensitivity to low Pi and low N during seedling stages (Figure S6A,B). Additionally, low N inhibited, whereas low Pi induced, the phosphorus starvation responses (Figure S7A–C), consistent with findings made in previous studies (Hu et al. 2019; Liu et al. 2025). Moreover, we noticed that loss of function of *TaPHL7* alleviates the repression of Pi‐response genes (*TaPHT1;3*, *TaPHT1;6*) and reduces the expression of nitrogen transport gene (*TaNRT2.1*) under low nitrogen conditions (Figure S7A,B,D), suggesting that *TaPHL7* may be a crucial node in the coordinated regulation of nitrogen and phosphorus utilisation. We next asked if *TaPHL7* played a role in regulating nitrogen–phosphorus signalling to regulate plant growth and development. As mentioned above, the expression of *TaPHL7* was marginally responsive to Pi (Figure S3A). However, downstream from *TaPHL7*, the expression of *TaPHT1;3* was induced by Pi and the response was substantially compromised in the *taphl7* mutants (Figure 5A). These results suggest that Pi‐regulated phosphorus signalling is genetically, at least in part, dependent on *TaPHL7*.

We then examined the response of the *taphl7* mutants to nitrate. In contrast to that of Pi, nitrate efficiently and rapidly induced the expression of *TaPHL7* (Figure 5B), suggesting that the transcription factor is capable of sensing and responding to a nitrate signal. Moreover, nitrate robustly induced the expression of *TaNRT2.1* and *TaNRT2.2*, two typical nitrate‐responsive genes, and the induction was remarkably amplified in the *taphl7* mutants (Figure 5C; Figure S5A). In the Pi signalling pathway, however, while nitrate also efficiently induced the expression of *TaPHT1;3* and *TaPHT1;9*, the induction was greatly reduced in the *taphl7* mutants (Figure 5D; Figure S5B). These results suggest that *TaPHL7* regulates both nitrogen and phosphorus signalling with distinctive mechanisms, by which nitrate may induce transcriptional derepression of *TaNRT2.1* and *TaNRT2.2* imposed by TaPHL7 but directly promotes the expression of *TaPHT1;3* and *TaPHT1;9*.

Given that *TaPHL7* regulates both nitrogen and phosphorus signalling, we noticed that the nitrate‐induced expression of the above‐mentioned genes was in a progressively delayed pattern, with peaks at 0.5 h for *TaPHL7*, 1–3 h for *TaNRT2.1* and *TaNRT2.2* and 3–6 h for *TaPHT1;3* and *TaPHT1;9* (Figure 5B–D; Figure S5A,B). This pattern implies a *TaPHL7*‐based sequential transcription derepression or activation mechanism to regulate nitrogen and phosphorus signalling. In agreement with this notion, multiple putative P1BS motifs were found in the promoters of genes related to nitrogen and phosphorus metabolism, including multiple *TaNRTs*, *TaGS1s*, *TaSPX3*, *TaPHTs* genes (Table S2). Collectively, these results suggest that *TaPHL7* regulates nitrogen and phosphorus utilisation.

As *TaPHL7* may regulate nitrogen and phosphorus utilisation, we explored its potentials in grain yield in a 2‐year field trial. As mentioned earlier, the *taphl7* mutants had an increased tiller number and an early‐maturing phenotype (see Figure 2E–G). We found that the grain number per spike was increased in the *taphl7* mutants (Figure 5E), consistent with the increase in spike length (Figure S5C,D). Compared with wild type, the grain length and grain width were increased and decreased in the *taphl7* mutants at varying degrees, respectively (Figure 5F,G; Figure S5E,F), eventually resulting in an unaltered or increased grain weight (Figure 5H). Notably, the increased or nearly unaltered grain weight of the *taphl7* mutants (Figure 5H) suggests that the early‐maturing phenotype does not have negative effects on grain filling. As expected, overexpression of *TaPHL7* caused a phenotype opposite to that of *taphl7* or marginal alterations (Figure 5E–H; Figure S5C–F). Overall, grain yield of the *taphl7* mutants increased by 7.1%–29.4% compared with wild‐type ZM7698, while it decreased by 27.6%–34.3% in *TaPHL7*‐overexpressing plants (Figure 5I). These results suggest that loss‐of‐function mutations in *TaPHL7* promote early maturation and increase grain yield under natural cultivation conditions.

### 2.6 *TaPHL7* Underwent Artificial Selection During Wheat Improvement

**FIGURE 6:**
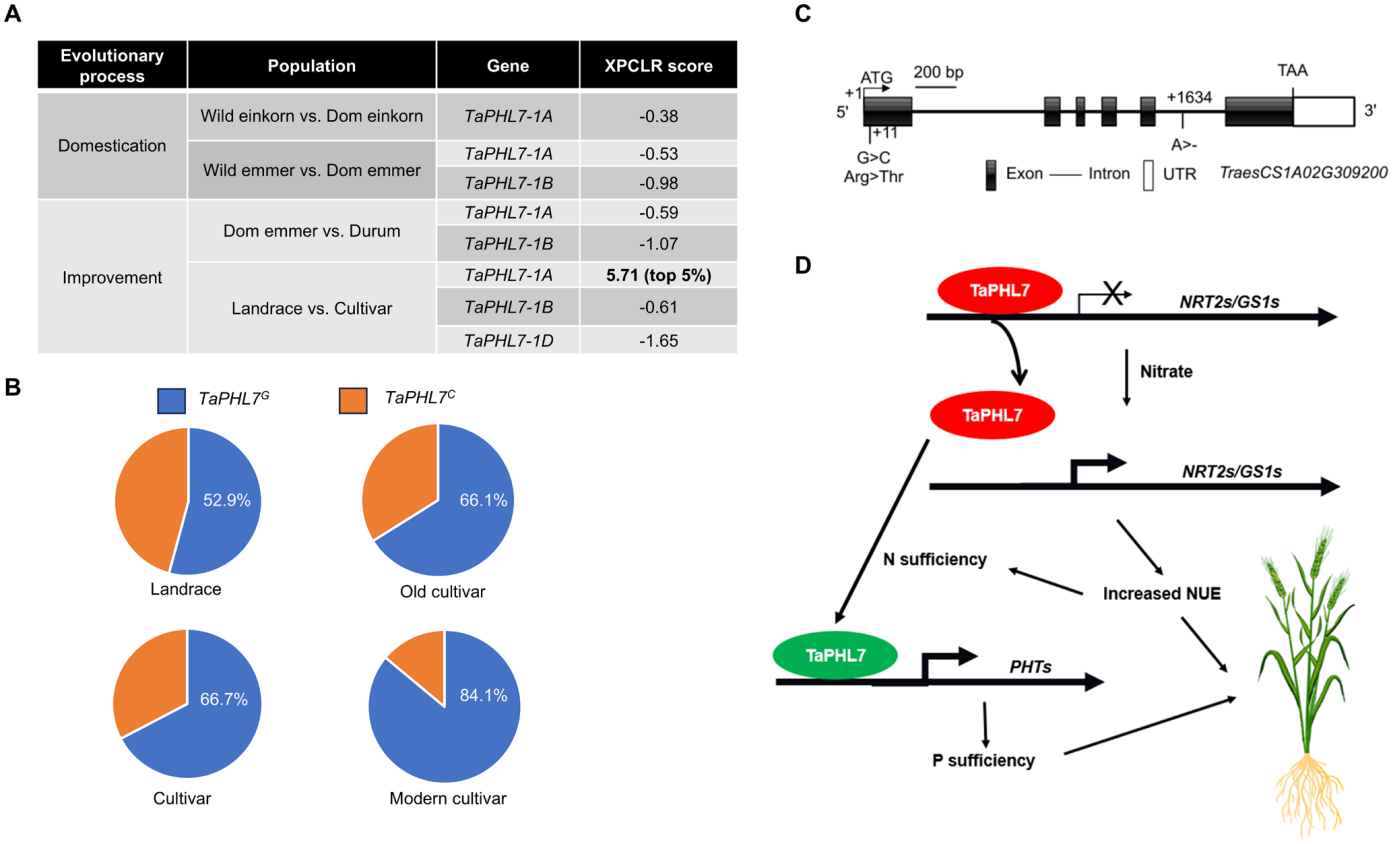
*TaPHL7* underwent artificial selection during wheat improvement. (A) Selection signal analysis of *TaPHL7* homologous genes during wheat domestication and improvement. (B) Distribution of two *TaPHL7* haplotypes in four wheat historical populations. (C) Structural scheme of the *TaPHL7‐1A* gene. A missense mutation (+11; G to C) in exon 1 and a 1‐bp deletion mutation (+1634; A to ‐) in intron 5 are shown, respectively. (D) A proposed model of coordinated utilisation of nitrogen and phosphorus by TaPHL7 transcription factor in wheat.

In crops, key genes regulating important agronomic traits have usually been selected during domestication and breeding. Hexaploid wheat ( *Triticum aestivum* L. subsp. *aestivum*, AABBDD), the most widely cultivated *Triticum* genus worldwide, was evolved from two sequential polyploidisation processes during domestication, eventually giving rise to the hexaploid species (Wang et al. 2023) (Figure S8A). Of those evolutionary events, the wild emmer was domesticated into the cultivated plants via human selection and was further improved and cultivated at present day, such as the durum wheat (Figure S8A). To clarify whether *TaPHL7* was subjected to human selection, we investigated the comprehensive ploidy levels, including diploid, tetraploid and hexaploid, between wild and domesticated einkorn (AA), wild and domesticated emmer (AABB) and domesticated emmer and durum (AABB), using the genome resequencing data of 414 samples (Zhao et al. 2023). Analysis of three indicated ploidy levels revealed that *TaPHL7‐1A* in diploid einkorn and *TaPHL7‐1A* and *TaPHL7‐1B* in tetraploid emmer and durum were not subjected to selection during domestication or breeding (Figure 6A). However, an obvious selection signal was detected in *TaPHL7‐1A* between landraces and improved cultivars (Figure 6A), suggesting that *TaPHL7‐1A* underwent artificial selection during the improvement of local varieties to modern cultivars in hexaploid wheat.

Based on the distinctive breeding status, wheat species are classified into wild progenitors, early domesticates, landraces and cultivars. To identify key nucleotide loci mediating the selection of *TaPHL7‐1A*, we further analysed nucleotide polymorphism in the genomic sequence of *TaPHL7* using a core collection of 290 wheat germplasm (Pont et al. 2019), including 85 landraces, 59 old cultivars, 102 more recent cultivars and 44 modern cultivars (Figure 6B; Figure S8B). Nucleotide polymorphism analysis identified the presence of a single nucleotide polymorphism (SNP) in exon 1 and an InDel in intron 5 (a single nucleotide deletion) in the *TaPHL7‐1A* genomic region (Figure 6C). No other mutations were identified in the coding sequences of *TaPHL7‐1A*. The significance of the InDel in intron 5 remained to be investigated. The SNP in exon 1 caused a G‐to‐C transition, resulting in the substitution of the fourth amino acid residue Arg with Thr (Figure 6C). An analysis of these two haplotypes revealed that the *TaPHL7* ^*C*^ allele was gradually abandoned during wheat improvement, showing an occurrence of 44.7% in landraces, 33.9% in old cultivars, 32.3% in more recent cultivars and 13.6% in modern cultivars, respectively, whereas the *TaPHL7* ^*G*^ allele presented a reverse pattern (Figure 6B; Figure S6B). In subgenome B, 5 missense mutations were identified in *TaPHL7‐1B*. However, no apparent difference in distribution frequency was observed. Because of missing data of *TaPHL7* in subgenome D, possible genetic variations in *TaPHL7‐1D* remained to be clarified. Nevertheless, these results suggest that *TaPHL7‐1A* has been subjected to artificial selection during wheat improvement.

## 3 Discussion

In this study, we find that TaPHL7 plays a dual role in regulating utilisation of nitrogen and phosphorus. TaPHL7 acts as a transcriptional repressor to negatively control the expression of a subset of nitrogen utilisation genes, including *TaNRT2.1*, *TaNRT2.2* and *TaGS1;3*. Moreover, TaPHL7 also functions as a transcription activator to promote the expression of *TaPHT* genes during phosphorus starvation responses. An altered nitrate level triggers the transcription derepression of *TaNRT2s* and *TaGS1;3* imposed by TaPHL7, leading to an increase in nitrogen utilisation. The increased nitrogen level, in turn, promotes the expression of *TaPHT* genes, which is, at least partly, dependent on *TaPHL7*. Thus, TaPHL7 may regulate the utilisation of nitrogen and phosphorus in response to fluctuating levels of these nutrients under variable growth conditions (Figure 6D).

In plants, the GS/GOGAT cycle plays a central role in nitrogen assimilation. Ammonium, a major form of inorganic nitrogen utilised by plants, can be directly absorbed from soil, reduced from nitrate, released via photorespiration and derived from protein turnover. Ammonium is then converted or assimilated into organic nitrogen, which is catalysed by differentiated localisation and specialised physiological functions of GS/GOGAT enzymes (Krapp 2015). In higher plants, both GS and GOGAT enzymes have evolved into two types, cytosolic GS1 or plastidic GS2, and ferredoxin‐ or NADH‐dependent GOGAT, respectively. In particular, the members of genes encoding the GS1 isoenzymes were mostly duplicated (three in wheat, three in rice, five in maize, three in barley and five in *Arabidopsis*) (Liu et al. 2022), implying their indispensable functions in assimilating ammonium. Physiologically, GS1‐modulated reassimilation of ammonium during grain filling is of particular interest in crops, a process largely determining grain filling and thus yield in cereals. *TaGS1;3* is specifically expressed in developing grain, similar to *OsGS1;3* in rice, *ZmGS1‐2* in maize and *HvGS1;3* in barley (Fujita et al. 2022; Goodall et al. 2013; Muhitch et al. 2002), while the underlying regulatory mechanism of these specialised orthologs remains unknown. It is somewhat unexpected that TaPHL7 represses the transcription of *TaGS1;3* by binding to the P1BS elements harbouring in its promoter, providing a line of evidence supporting the notion that Pi signalling directly regulates nitrogen assimilation. In addition, multiple copies of P1BS elements are also found in the promoters of other *TaGS* genes (1–2 in *TaGS1;1* and 2–3 in *TaGS1;2*) and nitrate transporter genes (2 in *TaNRT2.1*, 5 in *TaNRT2.2* and 2–3 in *TaNRT2.3*), implying that TaPHL7 may orchestrate nitrogen metabolism in response to different developmental and environmental cues. Indeed, loss‐of‐function mutations in *TaPHL7* simultaneously enhanced nitrogen uptake, assimilation and remobilisation at both the vegetative stage and post‐anthesis stage. These observations suggest that TaPHL7 plays a vital role in regulating nitrogen metabolism and utilisation by directly targeting *TaGS1;3* and, possibly, genes encoding other GS isoenzymes and nitrate transporter genes.

*PHR1* and *PHR1*‐like genes have been characterised as master transcription regulators in Pi signalling (Ren et al. 2012; Rubio et al. 2001; Tian et al. 2024; Zhou et al. 2008). Similarly, TaPHL7 also functions in mediating Pi signalling in wheat. In *Arabidopsis*, AtPHR1 functions in regulating the PSR, and phosphorus content in the roots of the *atphr1* mutant is significantly increased under Pi‐deficient conditions (Nilsson et al. 2007). However, loss‐of‐function mutations in *TaPHL7* result in no obvious effect on phosphorus content under Pi‐deficient conditions, possibly due to the functional redundancy of TaPHR1 (Wang et al. 2013). Nevertheless, the *taphl7* mutations significantly increase the nitrogen content under normal growth conditions, implying that TaPHL7 may have evolved differentiated functions in modulating nitrogen metabolism in addition to its role in Pi signalling. Consistent with this notion, AtPHR1 was also reported to serve as a convergent point for the crosstalk between Pi and other essential nutrients, including sulphate, zinc and iron (Bournier et al. 2013; Khan et al. 2014; Rouached et al. 2011). Moreover, while the *taphl7* mutation increased nitrogen content, the impaired Pi acquisition results in a significant reduction of biomass under Pi‐sufficient conditions at the seedling stage, similar to phenomena observed in the *atphr1* mutant, suggesting that the balance between phosphorus and nitrogen is critical for appropriate growth potential at the seedling stage.

Despite the reduction of biomass in the vegetative growth stage, loss‐of‐function mutations in *TaPHL7* substantially improve nitrogen reassimilation and remobilisation during grain filling and thus boost wheat yield under natural cultivation conditions, further suggesting primary roles of *TaPHL7*‐modulated nitrogen metabolism at the post‐anthesis stage. Consistently, acting as a negative regulator of nitrogen metabolism, the transcripts of *TaPHL7* are less abundant in developing grain and are progressively reduced during grain filling, thus releasing TaGS1;3 activity to reassimilate ammonium in grain. We notice that the spatiotemporal expression patterns of *TaPHL7* in other vegetative tissues/organs are consistent with those in developing grain, suggesting that *TaPHL7* may function as a switch controlling complete nitrogen metabolism at the grain‐filling stage. In agreement with this notion, loss‐of‐function mutations in *TaPHL7* substantially increased nitrogen remobilisation from leaves source to grain sink, resulting in earlier maturation and increased grain weight. Identification of components regulating the TaPHL7 activity regarding different developmental cues should shed light on the molecular mechanism of *TaPHL7*‐orchestrated nitrogen and phosphorus homeostasis.

Grain yield is the most important target for crop breeding, and the genetic locus controlling yield‐related traits is generally subjected to natural or artificial selection during domestication or breeding programmes. Ploidy level analysis shows that *TaPHL7‐1A* was subjected to artificial selection during the improvement of hexaploid bread wheat. Nucleotide polymorphism analysis reveals a gradually increased occurrence of the *TaPHL7* ^*G*^ haplotype during the course of wheat improvement. Generally, the breeding of high‐yield cultivars accompanied by the application of large amounts of nitrogen fertiliser, and the fixation of the *TaPHL7* ^*C*^ haplotype in modern cultivars suggest that *TaPHL7* ^*G*^ may represent an elite allele boosting nitrogen utilisation and yield. Given that the missense mutation of *TaPHL7* ^*G*^ results in a substitution mutation of a highly conserved amino acid residue of TaPHL7, it will be interesting to determine how this substitution alters the activity of the transcription factor.

## 4 Methods

### 4.1 Plant Materials and Growth Conditions

The wild‐type hexaploid wheat cultivar Zhengmai7698 (ZM7698) was used in this study unless indicated otherwise. For the hydroponic cultivation, seeds of the indicated genotypes were simultaneously surface‐sterilised using 2% sodium hypochlorite, imbibed for 1 day and grown in deionised water for 5 days, followed by being cultivated in a modified Hoagland hydroponic solution for 12 days. The modified solution contains 1 mM MgSO_4_, 1.5 mM CaCl_2_, 1.5 mM KCl, 100 μM Fe‐EDTA, 1 μM ZnSO_4_, 1 μM MnSO_4_, 1 μM H_3_BO_4_, 0.5 μM CuSO_4_ and 0.05 μM (NH_4_)_6_Mo_7_O_24_, supplying sufficient phosphate (control; 0.20 mM KH_2_PO_4_) or deficient phosphate (low Pi; 0.01 mM KH_2_PO_4_), or with sufficient nitrogen (control; 2.00 mM Ca(NO_3_)_2_) or deficient nitrogen (low N; 0.20 mM Ca(NO_3_)_2_), and the pH value was adjusted to 5.8. The seedlings were cultured in a greenhouse with 60%–70% relative humidity under 16 h/8 h light/dark and 22°C–25°C/16°C–18°C day/night conditions.

To generate the *TaPHL7*‐knockout and *TaPHL7*‐overexpressing plants, the p*CRISPR‐TaPHL7* vector and p*Ubi::TaPHL7‐OE* (see Plasmid Construction for details) vector were transformed into the callus of wild‐type ZM7698 via the *Agrobacterium*‐mediated transformation system, respectively. Genotypes of all *TaPHL7*‐edited plants were confirmed by sequencing, and three *CasΦ2* ^*Ta*^‐free null mutants were identified and characterised, designated as *taphl7‐1*, *taphl7‐2* and *taphl7‐3*, respectively. The positive transgenic lines of *TaPHL7*‐overexpressed plants were screened by a PAT/BAR Rapid Test Strip kit (Shanghai Youlong Biotech; cat. no. AA1032), and three *TaPHL7*‐overexpressed transgenic plants were adopted in this study, designated as *PHL7‐OX1*, *PHL7‐OX2* and *PHL7‐OX3*, respectively.

Field‐cultivated plants were grown during the routine wheat growing season under natural cultivation conditions at the Experimental Stations of Henan Agricultural University, Institute of Genetics and Developmental Biology, Chinese Academy of Sciences, and China Agricultural University, respectively. All experiments were conducted using a randomised block design (RBD), with each experimental group including three biological replicates. The experimental groups consisted of knockout plants, overexpressing plants and wild type. The planting configuration consisted of 2‐m‐long rows, with 10 rows per plot, a row spacing of 25 cm and a plant spacing of 5 cm with routine management. For the field trial, the compound fertiliser (750 kg/ha; the contents of nitrogen (N), phosphorus pentoxide (P_2_O_5_) and potassium oxide (K_2_O) in the compound fertiliser are ≥ 15%, 15% and 15%, respectively) was applied before sowing, and urea (240 kg/ha; *N* ≥ 46%) was applied at the jointing stage, respectively.

### 4.2 Plasmid Construction

The promoter fragment or coding sequence of *TaGS1;3* and *TaPHL7* used in the plasmid construction was PCR‐amplified using Yumai49 (YM49) genomic DNA or cDNA as templates unless indicated otherwise, and primer pairs embedded with appropriate restriction sites, and then inserted into the indicated vector via homologous recombination. Briefly, to construct a bait reporter of p*TaGS1;3p::AUR1‐C* used in the Y1H assay, a 2000‐bp fragment of the *TaGS1;3–4D* promoter was inserted into the *Sal*I/*Sac*I sites of a pAbAi vector (Clontech; 630 491). To construct the bacterial expression vector of p*T7::HIS‐TaPHL7* used in the EMSA assay, the coding sequence of *TaPHL7‐1A* was inserted into the *Bam*HI/*Eco*RI sites of a pET‐28a(+) vector (Novagen; 69 864–3). To construct the plant expression vector of p*35S::TaPHL7‐GFP* used in the ChIP‐qPCR analysis, the coding sequence (without stop codon) of *TaPHL7‐1A* was inserted into *Bam*HI/*Kpn*I sites of an improved pUC‐HA‐GFP vector (Ma et al. 2023). To construct a reporter plasmid of p*TaGS1;3p::LUC* used in the transient expression analysis, a 1796‐bp promoter fragment (−1 to −1796; the putative transcription start site is referred to as +1) of *TaGS1;3–4D* harbouring 6 of P1BS and 2 of P1BS‐like elements was inserted into the *Kpn*I/*Not*I sites of pGreenII 0800‐LUC vector (Hellens et al. 2005). A similar strategy was applied to generate a reporter harbouring a P4‐deleted (Δ4) or P5‐deleted (Δ5) promoter. Similarly, a 1000‐bp and 1536 bp fragment of the *TaPHT1;3‐5B* and *TaPHT1;9–4B* promoter was inserted into the *Bam*HI site of pGreenII 0800‐LUC to generate the reporter plasmid of p*TaPHT1;3::LUC* and p*TaPHT1;9p::LUC*, respectively. To construct an effector plasmid of p*35S::TaPHL7‐FLAG* used in the transient expression analysis, the coding sequence (without stop codon) of *TaPHL7‐1A* was inserted into *Xba*I/*Kpn*I sites of an improved pCAMBIA1300 vector (Yang et al. 2023). To construct the plant expression vector of p*Ubi::TaPHL7‐GFP* used in the subcellular localisation analysis, the coding sequence (without stop codon) of *TaPHL7‐1A* was inserted into *Hin*dIII/*Bam*HI sites of the pJIT163‐GFP vector. To construct a plant expression vector of p*Ubi::TaPHL7‐OE* used in the generation of *TaPHL7*‐overexpressing plants, the coding sequence of *TaPHL7‐1A* in ZM7698 was inserted into the *Sac*I site of a pLH5 vector (Zhao et al. 2024). To construct a *TaPHL7*‐editing vector of p*CRISPR‐TaPHL7*, two single guide RNAs (sgRNAs) were designed based on the conservation of *TaPHL7* in three subgenomes, designated as sgRNA1 and sgRNA2 (Figure 2A), respectively, and driven by the *TaU3* promoter harboured in a pCRISPR/CasΦ2^Ta^‐V3 vector (Zhao et al. 2024). All constructs were verified by extensive restriction digestion and DNA sequencing analysis, and primers used in the plasmid construction are listed in Table S3.

### 4.3 RT‐qPCR Analysis

Total RNA from various organs/tissues as indicated was extracted using an RNA Easy Fast Plant Tissue Kit (TIANGEN; cat. no. DP452) according to the manufacturer's instructions, and approximately 1 μg of total RNA was subjected to cDNA synthesis using the HiScript III RT SuperMix kit (Vazyme; cat. no. R323). qPCR experiments were performed with gene‐specific primers using ChamQ Blue Universal SYBR qPCR Master Mix (Vazyme; cat. no. Q312) in a real‐time system (BIO‐RAD; CFX96). The wheat *Actin* (*TraesCS1B02G283900*) or *TEF* (*TraesCS4D02G197100*) genes were used as an internal control for normalisation. Sequences of gene‐specific primers are listed in Table S3.

### 4.4 Yeast One‐Hybrid (Y1H) Assay

Y1H screening was performed using a Matchmaker Gold Yeast One‐Hybrid Library Screening System (Clontech; cat. no. 630491). The reporter vector p*TaGS1;3p::AUR1‐C* was introduced into Y1HGold to create a reporter strain. The cDNA library was generated using mixed samples of developing grain of Xiaoyan81, and was collected at 0‐, 5‐ and 10‐days post anthesis, respectively. Transformed cells were plated on an SD/−Leu/+AbA^100‐500 ng/mL^ media to select positive clones during both primary screening and the second round of point‐to‐point validation.

### 4.5 Electrophoretic Mobility Shift Assay (EMSA)

EMSA was performed as described previously (Wang, Su, et al. 2021) with minor modifications using a Chemiluminescent EMSA Kit (Beyotime; cat. no. GS009) following the manufacturer's instructions. Briefly, the p*T7::HIS‐TaPHL7* expression vector was transformed into the *E. coli* BL21 strain, and the purification of HIS‐TaPHL7 recombinant protein was performed using the Ni‐Agarose Resin (CWBIO; cat. no. CW0010S). A single‐stranded DNA oligonucleotide harbouring six P1BS elements or two P1BS‐mutated elements (listed in Figure 1D) was synthesised respectively and labelled with biotin using an EMSA Probe Biotin Labeling Kit (Beyotime; cat. no. GS008) and was further annealed to make the double‐stranded DNA probes. For the binding reaction, approximately 2 μg of protein and 200 pM (1×) of biotin‐labelled probes were incubated in 20 μL of reaction mixture for 30 min at room temperature. The mixtures were separated in a 6% (w/v) polyacrylamide gel in 0.5× TBE buffer (45 mM Tris, 45 mM boric acid, 1 mM EDTA, pH 8.3). The signal was visualised in a chemiluminescence imaging system (GE HealthCare; Amersham Imager 600).

### 4.6 Chromatin Immunoprecipitation‐Quantitative PCR (ChIP‐qPCR) Assay

Wheat protoplasts were prepared from leaves derived from 7–10‐day‐old ZM7698 seedlings as described previously (Zhang, Zhang, Li, Li, et al. 2021), and the p*35S::TaPHL7‐GFP* or p*35S::GFP* (negative control) expression vector was separately transformed into protoplasts via the PEG‐mediated transfection system. After transformation and incubation in the dark for 16–20 h at room temperature, the protoplast was collected in a pre‐cooled PBS buffer (137 mM NaCl, 10 mM phosphate, 2.7 mM KCl, pH 7.4), and then cross‐linked in a PBS buffer containing 1% formaldehyde for 10 min at 4°C. To stop the reaction, a PBS buffer containing 125 mM glycine was added, followed by incubation at room temperature for 5 min. After nuclear isolation and sonication, the sonicated chromatin fragments were immunoprecipitated using the Anti‐GFP Nanobody Agarose Beads (AlpaLifeBio; cat. no. KTSM1301). Primer pairs specific to P0 and fragments harbouring P1 to P6 (Figure 1B,G), fragments harbouring P3‐2 to P3‐4 and P9‐1 to P9‐4 (Figure 3I; Figure S3E) are listed in Table S3.

### 4.7 Transient Expression Assays

For the analysis of transcriptional repression activity of TaPHL7 to *TaGS1;3*, the effector vector of p*35S::TaPHL7‐FLAG* or p*35S::FLAG* (negative control) and reporter vector of p*TaGS1;3p::LUC* were transformed into *Agrobacterium tumefaciens* cells, respectively. Positive strains carrying effector or reporter were co‐infiltrated into tobacco leaves. After the infiltration, the plants were cultured at 22°C in the dark for 1 day, and then grown under light for 1 day. An appropriate amount of 1 mM luciferin was spread over the infiltrated leaves, followed by incubation in the dark for 10 min, and images of chemiluminescence were captured using an in vivo imaging system (Berthold; Night Shade LB985). A similar procedure was adopted to analyse the transcriptional activation of TaPHL7 to *TaPHT1;3* and *TaPHT1;9*. The luciferase activity was analysed using a Luciferase Reporter Gene Assay Kit (YEASEN; cat. no. 11401ES60) following the manufacturer's instructions.

For the analysis of subcellular localisation of TaPHL7, approximately 10 μg of p*Ubi::TaPHL7‐GFP* and p*Ubi::GFP* (negative control) expression vector was separately transformed into protoplasts via PEG‐mediated transfection and incubated in the dark at room temperature for 16 h. The protoplast was imaged using a confocal laser scanning microscope (Nikon; A1HD25).

### 4.8 Immunoblotting Analysis

To examine TaGS1;3 levels in the *taphl7* mutants and *TaPHL7*‐overexpressing plants, developing grain was collected at 16 days post anthesis and ground into fine powder in liquid nitrogen. Approximately 0.2 g of samples were suspended in 800 μL of cold extraction buffer (50 mM Tris, 150 mM NaCl, 1% Triton X‐100, 1% sodium deoxycholate, 0.1% SDS and 1 mM EDTA, pH 7.4), and the supernatant was collected by centrifugation at 12,000 *g* at 4°C for 10 min. Approximately 20 μg of protein sample was separated in 12% (w/v) polyacrylamide gel and electrically transferred onto a polyvinylidene difluoride membrane (0.45 μm), followed by the immunoblotting analysis. The TaGS1;3‐specific antibody (Wei et al. 2021) and a mouse monoclonal anti‐HSP82 antibody (Beijing Protein Innovation; cat. no. AbM51099‐31‐PU) were adopted to detect protein abundance, respectively. The signal was detected using a SuperSignal West Femto Maximum Sensitivity Substrate kit (Thermo Scientific; cat. no: 34096) according to the manufacturer's instructions, and visualised in an automatic chemiluminescence imaging system (Tanon Life Science; Tanon 5200).

### 4.9 Determination of Phosphorus and Nitrogen Content

Measurement of nitrogen and phosphorus contents was conducted as previously described (Chiou et al. 2006) with minor modifications. Briefly, roots of 18‐day‐old seedlings or vegetative organs and mature grains of field‐grown plants were collected and subjected to desiccation at 105°C for 30 min, followed by drying at 80°C for 3–5 days, and then ground into fine powder for subsequent analysis. Approximately 0.1 g of samples was homogenised in 5 mL of sulfuric acid for 24 h at room temperature, followed by incubation at 380°C for 1 h. A clear mixture was obtained via periodical addition of hydrogen peroxide, followed by a 400‐fold dilution, and then subjected to a continuous segmented flow analyser (SEAL Analytical; SEAL AutoAnalyzer3 HR).

Content of remobilised nitrogen was determined by the ratio of remobilised nitrogen during 40 days post‐anthesis to total nitrogen in vegetative organs at anthesis. NRE was defined as the ratio of nitrogen remobilised from senescence organs to nitrogen in grain (Xu et al. 2012), and was determined by a formula: NRE = (nitrogen content of vegetative organs at anthesis—nitrogen content of vegetative organs at mature stage) ÷ nitrogen content of mature grain.

To examine nitrate or ammonium content in developing grain, samples were collected at 16 days post anthesis and then ground into fine powder in liquid nitrogen. For the nitrate content analysis, approximately 0.2 g of sample was suspended in 20 mL of 10% trichloroacetic acid, followed by homogenisation at room temperature for 20 min. Then, the mixture was centrifuged at 12,000 *g* for 15 min, and 500 μL of the supernatant aliquot was collected and mixed with 300 μL of sulfuric acid solution containing 0.36 μM salicylic acid, followed by incubation at room temperature for 20 min. The mixture was gently diluted with 7 mL of 2 M NaOH solution and cooled at room temperature for the spectrophotometrical analysis. For the ammonium content analysis, approximately 0.2 g of sample was suspended in 1 mL of 10% acetic acid solution and incubated at 4°C for 15 min. After centrifugation at 12,000 *g* for 15 min at 4°C, 800 μL of the supernatant aliquot was collected and sequentially mixed with 7 mL of distilled water, 1 mL of chromogenic reagent (374.76 mM C_7_H_5_NaO_3_ and 237.83 mM C_4_H_4_O_6_K_2_), 200 μL of sodium hypochlorite solution (6.73 mL of 5% NaClO and 31.19 mL of 2 M NaOH mixed in 100 mL of ddH_2_O) and 200 μL of 33.56 mM sodium nitroprusside solution, followed by incubation at room temperature for 1 h. Finally, 200 μL of the reaction mixtures was aliquoted and subjected to a multi‐wavelength microplate reader (Molecular Devices; SpectraMax M2). The concentration of nitrate and ammonium was calculated according to the absorbance value determined at 410 nm and 697 nm, respectively (Wei et al. 2018).

To examine nitrate uptake rate and root‐to‐shoot translocation activity, 16‐day‐old wheat seedlings grown under a modified Hoagland solution containing 2 mM KNO_3_ were rinsed in 0.1 mM CaSO_4_ solution for 1 min, and then cultured in a fresh Hoagland solution containing 2 mM ^15^N‐labelled KNO_3_ (98 atom % ^15^N; Sigma; cat. no. 335134‐1G) for 3 h, followed by rinsing in 0.1 mM CaSO_4_ solution for 1 min. Then, roots and shoots of 6 seedlings were collected and subjected to desiccation at 105°C for 30 min, followed by drying at 80°C for 3–5 days, and further ground into fine powder. To examine ^15^N content in the collected organs, approximately 0.1 g of sample was subjected to an isotope ratio mass spectrometer (Thermo Fisher Scientific; Finnigan Delta Plus XP) with an elemental analyser (Thermo Fisher Scientific; Flash EA 1112). Nitrate uptake rate was defined as total absorbed ^15^N per dry weight of seedling per hour, while nitrate translocation activity was calculated as the ratio of ^15^N content of shoots to ^15^N content of roots (Liu et al. 2016).

### 4.10 Analysis of the GS Activity

To examine the GS activity of the *taphl7* mutants, developing grain was collected at 16 days post anthesis and was ground into fine powder in liquid nitrogen. Approximately 0.5 g of sample was suspended in 1.5 mL of extraction buffer (100 mM Tris–HCl, 2 mM MgCl_2_, 1 mM EDTA, 0.1% β‐mercaptoethanol, 1 mM PMSF, pH 7.6) and then was gently homogenised at 4°C for 20 min. The supernatant was collected by centrifugation at 13000 *g* for 30 min at 4°C, and 600 μL of aliquot was sequentially mixed with 600 μL of 250 mM imidazole buffer (pH 7.0), 400 μL of 300 mM sodium glutamate solution (pH 7.0), 400 μL of 30 mM ATP‐Na_2_ (pH 7.0) and 200 μL of 500 mM magnesium sulphate solution, followed by incubation at 25°C for 5 min. Then, the enzymatic reaction began upon addition of 200 μL of 1 M hydroxylamine fresh solution and lasted for 15 min at 25°C, followed by addition of 800 μL of stop solution (a balanced mixture of 10% FeCl_3_, 24% C_2_HCl_3_O_2_ and 50% HCl). After centrifugation at 10 000 *g* for 5 min at room temperature, 200 μL of supernatant aliquot was collected and subjected to a multi‐wavelength microplate reader (Molecular Devices; SpectraMax M2). The total GS activity was spectrophotometrically measured by recording the rate of γ‐glutamylhydroxamate synthesis at 540 nm and was defined as micromoles of γ‐glutamylhydroxamate synthesised (25°C, pH 7.6) per hour per fresh weight of grain (Wei et al. 2020). The in‐gel activity assay was performed as previously described (Wei et al. 2020).

### 4.11 Selective Sweep Detection in Wheat

A cross‐population composite likelihood ratio (XP‐CLR v1.0) was used to detect genomic regions under selection (Chen et al. 2010). XP‐CLR scores were computed between population pairs using the parameters ‐w1 0.005500 10 000 ‐p1 0.95. Genetic distances were estimated based on recombination rate data from a previously published study (International Wheat Genome Sequencing 2018). To normalise the XP‐CLR statistics and detect the boundary of genomic regions, we applied the GenWin R package (https://cran.r‐project.org/web/packages/GenWin), using a smoothness value of 2000 and method 4. Regions falling within the top 5% of XP‐CLR values for each calculation were considered candidate selective sweeps, with different thresholds determined for each subgenome.

## Author Contributions

H.W. and Z.Z. performed most of the experiments, assisted by Y.G., Xiaohui M. and J.N. X.W., S.X., X.L., Y.Y. and Z.N. provided experimental materials and facilities. J.Z., Xinming M., F.L. and H.W. designed the study and analysed the data. J.Z., H.W. and Q.W. analysed the data and wrote the manuscript. All authors read and approved the manuscript.

## Funding

This work was supported by the State Key Laboratory of Plant Genomics (SKLPG2023‐22), the Ministry of Science and Technology of the People's Republic of China (2021YFD1700900, 2022YFD1201700) and the National Natural Science Foundation of China (32071956, 32330010).

## Conflicts of Interest

The authors declare no conflicts of interest.

## Supporting information


**Figure S1:** Transcriptional profiles and repression of *TaGS1;3* by TaPHL7.
**Figure S2:** Phylogenetic analysis of TaPHR and TaPHL proteins.
**Figure S3:** *TaPHL7* regulates Pi signalling and Pi acquisition.
**Figure S4:** *TaPHL7* regulates nitrogen metabolism.
**Figure S5:** *TaPHL7* controls maturity and grain yield.
**Figure S6:** Responses of the *taphl7* mutants to low nitrogen and low phosphorus.
**Figure S7:** Regulation of nitrogen and phosphorus marker genes by *TaPHL7*.
**Figure S8:** Evolution of *TaPHL7* during wheat domestication and improvement.
**Table S1:** Candidate TaGS1;3‐interacting proteins identified by Y1H.
**Table S2:** Candidate is of P1BS motifs in the promoter of genes regulating nitrogen and phosphorus metabolism.
**Table S3:** Primers used in this study.

## Data Availability

The data that support the findings of this study are available in the Supporting Information of this article.

## References

[pbi70493-bib-0001] Bournier, M. , N. Tissot , S. Mari , et al. 2013. “ *Arabidopsis* Ferritin 1 (*AtFer1*) Gene Regulation by the Phosphate Starvation Response 1 (AtPHR1) Transcription Factor Reveals a Direct Molecular Link Between Iron and Phosphate Homeostasis.” Journal of Biological Chemistry 288: 22670–22680. 23788639 10.1074/jbc.M113.482281 PMC3829352

[pbi70493-bib-0002] Chen, H. , N. Patterson , and D. Reich . 2010. “Population Differentiation as a Test for Selective Sweeps.” Genome Research 20: 393–402. 20086244 10.1101/gr.100545.109 PMC2840981

[pbi70493-bib-0003] Chen, J. , Y. Wang , F. Wang , et al. 2015. “The Rice CK2 Kinase Regulates Trafficking of Phosphate Transporters in Response to Phosphate Levels.” Plant Cell 27: 711–723. 25724641 10.1105/tpc.114.135335 PMC4558666

[pbi70493-bib-0004] Chiou, T.‐J. , K. Aung , S.‐I. Lin , C.‐C. Wu , S.‐F. Chiang , and C.‐l. Su . 2006. “Regulation of Phosphate Homeostasis by microRNA in *Arabidopsis* .” Plant Cell 18: 412–421. 16387831 10.1105/tpc.105.038943 PMC1356548

[pbi70493-bib-0028] de Magalhães, J. V. , V. M. C. Alves , R. F. de Novais , et al. 1998. “Nitrate Uptake by Corn Under Increasing Periods of Phosphorus Starvation.” Journal of Plant Nutrition 21: 1753–1763.

[pbi70493-bib-0005] Fujita, T. , M. P. Beier , M. Tabuchi‐Kobayashi , et al. 2022. “Cytosolic Glutamine Synthetase GS1;3 Is Involved in Rice Grain Ripening and Germination.” Frontiers in Plant Science 13: 835835. 35211144 10.3389/fpls.2022.835835 PMC8861362

[pbi70493-bib-0006] Goodall, A. J. , P. Kumar , and A. K. Tobin . 2013. “Identification and Expression Analyses of Cytosolic Glutamine Synthetase Genes in Barley (*Hordeum vulgare* L.).” Plant and Cell Physiology 54: 492–505. 23324171 10.1093/pcp/pct006

[pbi70493-bib-0007] Grün, A. , P. Buchner , M. R. Broadley , and M. J. Hawkesford . 2018. “Identification and Expression Profiling of Pht1 Phosphate Transporters in Wheat in Controlled Environments and in the Field.” Plant Biology 20: 374–389. 29148171 10.1111/plb.12668 PMC5887882

[pbi70493-bib-0008] Guo, H.‐L. , M.‐Z. Tian , X. Ri , and Y.‐F. Chen . 2025. “Phosphorus Acquisition, Translocation, and Redistribution in Maize.” Journal of Genetics and Genomics 52: 287–296. 39389460 10.1016/j.jgg.2024.09.018

[pbi70493-bib-0009] He, X. , B. Qu , W. Li , et al. 2015. “The Nitrate‐Inducible NAC Transcription Factor TaNAC2‐5A Controls Nitrate Response and Increases Wheat Yield.” Plant Physiology 169: 1991–2005. 26371233 10.1104/pp.15.00568 PMC4634051

[pbi70493-bib-0010] Hellens, R. P. , A. C. Allan , E. N. Friel , et al. 2005. “Transient Expression Vectors for Functional Genomics, Quantification of Promoter Activity and RNA Silencing in Plants.” Plant Methods 1: 13. 16359558 10.1186/1746-4811-1-13 PMC1334188

[pbi70493-bib-0011] Hu, B. , Z. Jiang , W. Wang , et al. 2019. “Nitrate–NRT1.1B–SPX4 Cascade Integrates Nitrogen and Phosphorus Signalling Networks in Plants.” Nature Plants 5: 401–413. 30911122 10.1038/s41477-019-0384-1

[pbi70493-bib-0012] International Wheat Genome Sequencing . 2018. “Shifting the Limits in Wheat Research and Breeding Using a Fully Annotated Reference Genome.” Science 361: eaar7191. 30115783 10.1126/science.aar7191

[pbi70493-bib-0013] Jia, X. , L. Wang , L. Nussaume , and K. Yi . 2023. “Cracking the Code of Plant Central Phosphate Signaling.” Trends in Plant Science 28: 267–270. 36588035 10.1016/j.tplants.2022.12.008

[pbi70493-bib-0014] Kant, S. , M. Peng , and S. J. Rothstein . 2011. “Genetic Regulation by NLA and microRNA827 for Maintaining Nitrate‐Dependent Phosphate Homeostasis in *Arabidopsis* .” PLoS Genetics 7: e1002021. 21455488 10.1371/journal.pgen.1002021 PMC3063762

[pbi70493-bib-0015] Khan, G. A. , S. Bouraine , S. Wege , et al. 2014. “Coordination Between Zinc and Phosphate Homeostasis Involves the Transcription Factor PHR1, the Phosphate Exporter PHO1, and Its Homologue PHO1;H3 in *Arabidopsis* .” Journal of Experimental Botany 65: 871–884. 24420568 10.1093/jxb/ert444 PMC3924728

[pbi70493-bib-0016] Krapp, A. 2015. “Plant Nitrogen Assimilation and Its Regulation: A Complex Puzzle With Missing Pieces.” Current Opinion in Plant Biology 25: 115–122. 26037390 10.1016/j.pbi.2015.05.010

[pbi70493-bib-0017] Li, J. , H. Cao , S. Li , et al. 2025. “Genetic and Molecular Mechanisms Underlying Nitrogen Use Efficiency in Maize.” Journal of Genetics and Genomics 52: 276–286. 39515641 10.1016/j.jgg.2024.10.007

[pbi70493-bib-0018] Li, S. , Y. Tian , K. Wu , et al. 2018. “Modulating Plant Growth‐Metabolism Coordination for Sustainable Agriculture.” Nature 560: 595–600. 30111841 10.1038/s41586-018-0415-5 PMC6155485

[pbi70493-bib-0019] Lin, W.‐Y. , T.‐K. Huang , and T.‐J. Chiou . 2013. “NITROGEN LIMITATION ADAPTATION, a Target of microRNA827, Mediates Degradation of Plasma Membrane‐Localized Phosphate Transporters to Maintain Phosphate Homeostasis in *Arabidopsis* .” Plant Cell 25: 4061–4074. 24122828 10.1105/tpc.113.116012 PMC3877804

[pbi70493-bib-0020] Liu, B. , W. Xu , Y. Niu , et al. 2025. “TaTCP6 Is Required for Efficient and Balanced Utilization of Nitrate and Phosphorus in Wheat.” Nature Communications 16: 1683. 10.1038/s41467-025-57008-0 PMC11830803 39956820

[pbi70493-bib-0021] Liu, F. , Z. Wang , H. Ren , et al. 2010. “OsSPX1 Suppresses the Function of OsPHR2 in the Regulation of Expression of *OsPT2* and Phosphate Homeostasis in Shoots of Rice.” Plant Journal 62: 508–517. 10.1111/j.1365-313X.2010.04170.x 20149131

[pbi70493-bib-0022] Liu, N. , W. Shang , M. Guan , et al. 2024. “Phosphate Deficiency Responsive TaSPX3 Is Involved in the Regulation of Shoot Phosphorus in *Arabidopsis* Plants.” Plant Physiology and Biochemistry 206: 108215. 38029619 10.1016/j.plaphy.2023.108215

[pbi70493-bib-0023] Liu, X. , B. Hu , and C. Chu . 2022. “Nitrogen Assimilation in Plants: Current Status and Future Prospects.” Journal of Genetics and Genomics 49: 394–404. 34973427 10.1016/j.jgg.2021.12.006

[pbi70493-bib-0024] Liu, Y. , B. Hu , and C. Chu . 2016. “ ^15^N‐Nitrate Uptake Activity and Root‐To‐Shoot Transport Assay in Rice.” Bio‐Protocol 6: e1897.

[pbi70493-bib-0025] Lv, Q. , Y. Zhong , Y. Wang , et al. 2014. “SPX4 Negatively Regulates Phosphate Signaling and Homeostasis Through Its Interaction With PHR2 in Rice.” Plant Cell 26: 1586–1597. 24692424 10.1105/tpc.114.123208 PMC4036573

[pbi70493-bib-0026] Ma, X. , J. Nian , H. Yu , et al. 2023. “Linking Glucose Signaling to Nitrogen Utilization by the OsHXK7‐ARE4 Complex in Rice.” Developmental Cell 58: 1489–1501. 37413992 10.1016/j.devcel.2023.06.003

[pbi70493-bib-0027] Maeda, Y. , M. Konishi , T. Kiba , et al. 2018. “A NIGT1‐Centred Transcriptional Cascade Regulates Nitrate Signalling and Incorporates Phosphorus Starvation Signals in *Arabidopsis* .” Nature Communications 9: 1376. 10.1038/s41467-018-03832-6 PMC5893545 29636481

[pbi70493-bib-0029] Medici, A. , A. Marshall‐Colon , E. Ronzier , et al. 2015. “AtNIGT1/HRS1 Integrates Nitrate and Phosphate Signals at the *Arabidopsis* Root Tip.” Nature Communications 6: 6274. 10.1038/ncomms7274 PMC4373655 25723764

[pbi70493-bib-0030] Muhitch, M. J. , H. Liang , R. Rastogi , and K. G. Sollenberger . 2002. “Isolation of a Promoter Sequence From the Glutamine synthetase1–2 Gene Capable of Conferring Tissue‐Specific Gene Expression in Transgenic Maize.” Plant Science 163: 865–872.

[pbi70493-bib-0031] Nilsson, L. , R. MÜLler , and T. H. Nielsen . 2007. “Increased Expression of the MYB‐Related Transcription Factor, *PHR1*, Leads to Enhanced Phosphate Uptake in *Arabidopsis thaliana* .” Plant, Cell & Environment 30: 1499–1512. 10.1111/j.1365-3040.2007.01734.x 17927693

[pbi70493-bib-0032] Peng, M. , C. Hannam , H. Gu , Y.‐M. Bi , and S. J. Rothstein . 2007. “A Mutation in *NLA*, Which Encodes a RING‐Type Ubiquitin Ligase, Disrupts the Adaptability of *Arabidopsis* to Nitrogen Limitation.” Plant Journal 50: 320–337. 10.1111/j.1365-313X.2007.03050.x 17355433

[pbi70493-bib-0033] Pont, C. , T. Leroy , M. Seidel , et al. 2019. “Tracing the Ancestry of Modern Bread Wheats.” Nature Genetics 51: 905–911. 31043760 10.1038/s41588-019-0393-z

[pbi70493-bib-0034] Ren, F. , Q. Q. Guo , L. L. Chang , et al. 2012. “ *Brassica napus PHR1* Gene Encoding a MYB‐Like Protein Functions in Response to Phosphate Starvation.” PLoS One 7: e44005. 22952851 10.1371/journal.pone.0044005 PMC3430610

[pbi70493-bib-0035] Rouached, H. , D. Secco , B. Arpat , and Y. Poirier . 2011. “The Transcription Factor PHR1 Plays a Key Role in the Regulation of Sulfate Shoot‐To‐Root Flux Upon Phosphate Starvation in *Arabidopsis* .” BMC Plant Biology 11: 19. 21261953 10.1186/1471-2229-11-19 PMC3036608

[pbi70493-bib-0036] Rubio, V. , F. Linhares , R. Solano , et al. 2001. “A Conserved MYB Transcription Factor Involved in Phosphate Starvation Signaling Both in Vascular Plants and in Unicellular Algae.” Genes & Development 15: 2122–2133. 11511543 10.1101/gad.204401 PMC312755

[pbi70493-bib-0037] Rufty, T. W. , C. T. Mackown , and D. W. Israel . 1990. “Phosphorus Stress Effects on Assimilation of Nitrate.” Plant Physiology 94: 328–333. 16667705 10.1104/pp.94.1.328 PMC1077228

[pbi70493-bib-0038] Rufty, T. W. , M. Y. Siddiqi , A. D. M. Glass , and T. J. Ruth . 1991. “Altered 13NO3− Influx in Phosphorus Limited Plants.” Plant Science 76: 43–48.

[pbi70493-bib-0039] Rufty, T. W. J. , D. W. Israel , R. J. Volk , J. Qiu , and T. Sa . 1993. “Phosphate Regulation of Nitrate Assimilation in Soybean.” Journal of Experimental Botany 44: 879–891.

[pbi70493-bib-0040] Teng, W. , Y. Y. Zhao , X. Q. Zhao , et al. 2017. “Genome‐Wide Identification, Characterization, and Expression Analysis of PHT1 Phosphate Transporters in Wheat.” Frontiers in Plant Science 8: 543. 28443126 10.3389/fpls.2017.00543 PMC5386973

[pbi70493-bib-0041] Tian, M.‐Z. , H.‐F. Wang , Y. Tian , et al. 2024. “ZmPHR1 Contributes to Drought Resistance by Modulating Phosphate Homeostasis in Maize.” Plant Biotechnology Journal 22: 3085–3098. 39037027 10.1111/pbi.14431 PMC11500998

[pbi70493-bib-0042] Wang, J. , J. Sun , J. Miao , et al. 2013. “A Phosphate Starvation Response Regulator ta‐PHR1 Is Involved in Phosphate Signalling and Increases Grain Yield in Wheat.” Annals of Botany 111: 1139–1153. 23589634 10.1093/aob/mct080 PMC3662521

[pbi70493-bib-0043] Wang, Q. , Q. Su , J. Nian , et al. 2021. “The Ghd7 Transcription Factor Represses *ARE1* Expression to Enhance Nitrogen Utilization and Grain Yield in Rice.” Molecular Plant 14: 1012–1023. 33930508 10.1016/j.molp.2021.04.012

[pbi70493-bib-0044] Wang, X. , H. F. Wang , Y. Chen , M. M. Sun , Y. Wang , and Y. F. Chen . 2020. “The Transcription Factor NIGT1.2 Modulates Both Phosphate Uptake and Nitrate Influx During Phosphate Starvation in *Arabidopsis* and Maize.” Plant Cell 32: 3519–3534. 32958562 10.1105/tpc.20.00361 PMC7610294

[pbi70493-bib-0045] Wang, Y. , Y.‐F. Chen , and W.‐H. Wu . 2021. “Potassium and Phosphorus Transport and Signaling in Plants.” Journal of Integrative Plant Biology 63: 34–52. 33325114 10.1111/jipb.13053

[pbi70493-bib-0046] Wang, Z. , L. Miao , Y. Chen , et al. 2023. “Deciphering the Evolution and Complexity of Wheat Germplasm From a Genomic Perspective.” Journal of Genetics and Genomics 50: 846–860. 37611848 10.1016/j.jgg.2023.08.002

[pbi70493-bib-0047] Wei, Y. , A. Shi , X. Jia , et al. 2018. “Nitrogen Supply and Leaf Age Affect the Expression of *TaGS1* or *TaGS2* Driven by a Constitutive Promoter in Transgenic Tobacco.” Genes 9: 406. 30103455 10.3390/genes9080406 PMC6115907

[pbi70493-bib-0048] Wei, Y. , X. Wang , Z. Zhang , et al. 2020. “Nitrogen Regulating the Expression and Localization of Four Glutamine Synthetase Isoforms in Wheat (*Triticum aestivum* L.).” International Journal of Molecular Sciences 21, no. 17: 6299. 32878133 10.3390/ijms21176299 PMC7504200

[pbi70493-bib-0049] Wei, Y. , S. Xiong , Z. Zhang , et al. 2021. “Localization, Gene Expression, and Functions of Glutamine Synthetase Isozymes in Wheat Grain (*Triticum aestivum* L.).” Frontiers in Plant Science 12: 580405. 33633754 10.3389/fpls.2021.580405 PMC7901976

[pbi70493-bib-0050] Wild, R. , R. Gerasimaite , J.‐Y. Jung , et al. 2016. “Control of Eukaryotic Phosphate Homeostasis by Inositol Polyphosphate Sensor Domains.” Science 352: 986–990. 27080106 10.1126/science.aad9858

[pbi70493-bib-0051] Xu, G. , X. Fan , and A. J. Miller . 2012. “Plant Nitrogen Assimilation and Use Efficiency.” Annual Review of Plant Biology 63: 153–182. 10.1146/annurev-arplant-042811-105532 22224450

[pbi70493-bib-0052] Xu, L. , H. Zhao , R. Wan , et al. 2019. “Identification of Vacuolar Phosphate Efflux Transporters in Land Plants.” Nature Plants 5: 84–94. 30626920 10.1038/s41477-018-0334-3

[pbi70493-bib-0053] Xu, L. L. , M. Q. Cui , C. Xu , et al. 2024. “A Clade of Receptor‐Like Cytoplasmic Kinases and 14–3‐3 Proteins Coordinate Inositol Hexaphosphate Accumulation.” Nature Communications 15: 5107. 10.1038/s41467-024-49102-6 PMC11178898 38877001

[pbi70493-bib-0054] Yamaji, N. , Y. Takemoto , T. Miyaji , N. Mitani‐Ueno , K. T. Yoshida , and J. F. Ma . 2017. “Reducing Phosphorus Accumulation in Rice Grains With an Impaired Transporter in the Node.” Nature 541: 92–95. 28002408 10.1038/nature20610

[pbi70493-bib-0055] Yang, J. , X. Qu , T. Li , et al. 2023. “HY5‐HDA9 Orchestrates the Transcription of HsfA2 to Modulate Salt Stress Response in *Arabidopsis* .” Journal of Integrative Plant Biology 65: 45–63. 36165397 10.1111/jipb.13372

[pbi70493-bib-0061] Zeng, H. , F. Chen , Q. Zhu , et al. 2025. “The Interplay Between Phosphorus Nutrition and Abiotic Stresses in Plants.” Journal of Genetics and Genomics 52, no. 12: 1507–1523. 10.1016/j.jgg.2025.08.008. 40850502

[pbi70493-bib-0056] Zhang, J. , H. Zhang , S. Li , J. Li , L. Yan , and L. Xia . 2021. “Increasing Yield Potential Through Manipulating of an *ARE1* Ortholog Related to Nitrogen Use Efficiency in Wheat by CRISPR/Cas9.” Journal of Integrative Plant Biology 63: 1649–1663. 34270164 10.1111/jipb.13151

[pbi70493-bib-0057] Zhang, S. , Y. Zhang , K. Li , et al. 2021. “Nitrogen Mediates Flowering Time and Nitrogen Use Efficiency via Floral Regulators in Rice.” Current Biology 31: 671–683. 33278354 10.1016/j.cub.2020.10.095

[pbi70493-bib-0058] Zhao, S. , X. Han , Y. Zhu , et al. 2024. “CRISPR/CasΦ2‐Mediated Gene Editing in Wheat and Rye.” Journal of Integrative Plant Biology 66: 638–641. 38351739 10.1111/jipb.13624

[pbi70493-bib-0059] Zhao, X. , Y. Guo , L. Kang , et al. 2023. “Population Genomics Unravels the Holocene History of Bread Wheat and Its Relatives.” Nature Plants 9: 403–419. 36928772 10.1038/s41477-023-01367-3

[pbi70493-bib-0060] Zhou, J. , F. Jiao , Z. Wu , et al. 2008. “ *OsPHR2* Is Involved in Phosphate‐Starvation Signaling and Excessive Phosphate Accumulation in Shoots of Plants.” Plant Physiology 146: 1673–1686. 18263782 10.1104/pp.107.111443 PMC2287342

