# Supplementary material for: TaPHL7 Transcription Factor Regulates Utilisation of Nitrogen and Phosphorus in Wheat
Source: Plant Biotechnol J. 2025 Dec 19;24(4):2522–37. doi: 10.1111/pbi.70493 (PMC13140452; doi:10.1111/pbi.70493)
Supplement: Supplementary file 1 — Figure S1: Transcriptional profiles and repression of TaGS1;3 by TaPHL7. Figure S2: Phylogenetic analysis of TaPHR and TaPHL proteins. Figure S3: TaPHL7 regulates Pi signalling and Pi acquisition. Figure S4: TaPHL7 regulates nitrogen metabolism. Figure S5: TaPHL7 controls maturity and grain yield. Figure S6: Responses of the taphl7 mutants to low nitrogen and low phosphorus. Figure S7: Regulation of nitrogen and phosphorus marker genes by TaPHL7. Figure S8: Evolution of TaPHL7 during wheat domestication and improvement. Table S1: Candidate TaGS1;3‐interacting proteins identified by Y1H. Table S2: Candidate is of P1BS motifs in the promoter of genes regulating nitrogen and phosphorus metabolism. Table S3: Primers used in this study. [file PBI-24-2522-s001.docx]

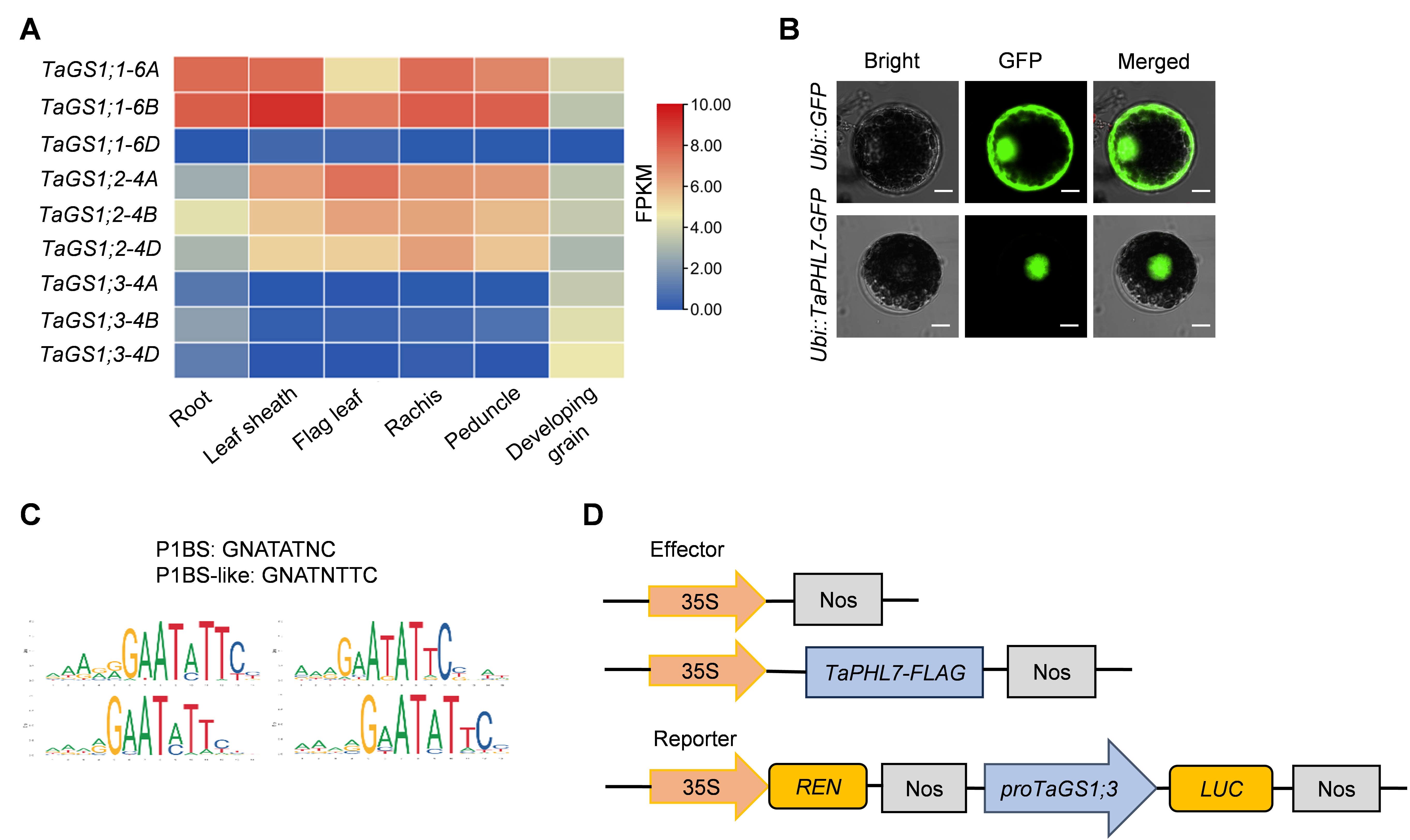


**Supplementary Fig. 1 Transcriptional profiles and repression of *TaGS1;3* by TaPHL7. Related to Figure 1.**

**(A)** Expression heat map of three *TaGS1* genes in the indicated organs and tissues at 16-day post anthesis.

**(B)** Subcellular localization of TaPHL7-GFP. Chemiluminescence images were captured from wheat protoplasts expressing a *Ubi::GFP* or a *Ubi::TaPHL7-GFP* transgene. Scale bars, 10 μm.

**(C)** Putative *cis*-elements of P1BS and P1BS-like for TaPHL7 binding predicted using a JASPAR database (<https://jaspar.elixir.no>).

**(D)** Diagrams of the effector and reporter constructs used in the transient expression assay in tobacco leaves. *REN* and *LUC* refer to the *Renilla* luciferase and firefly luciferase genes, respectively.


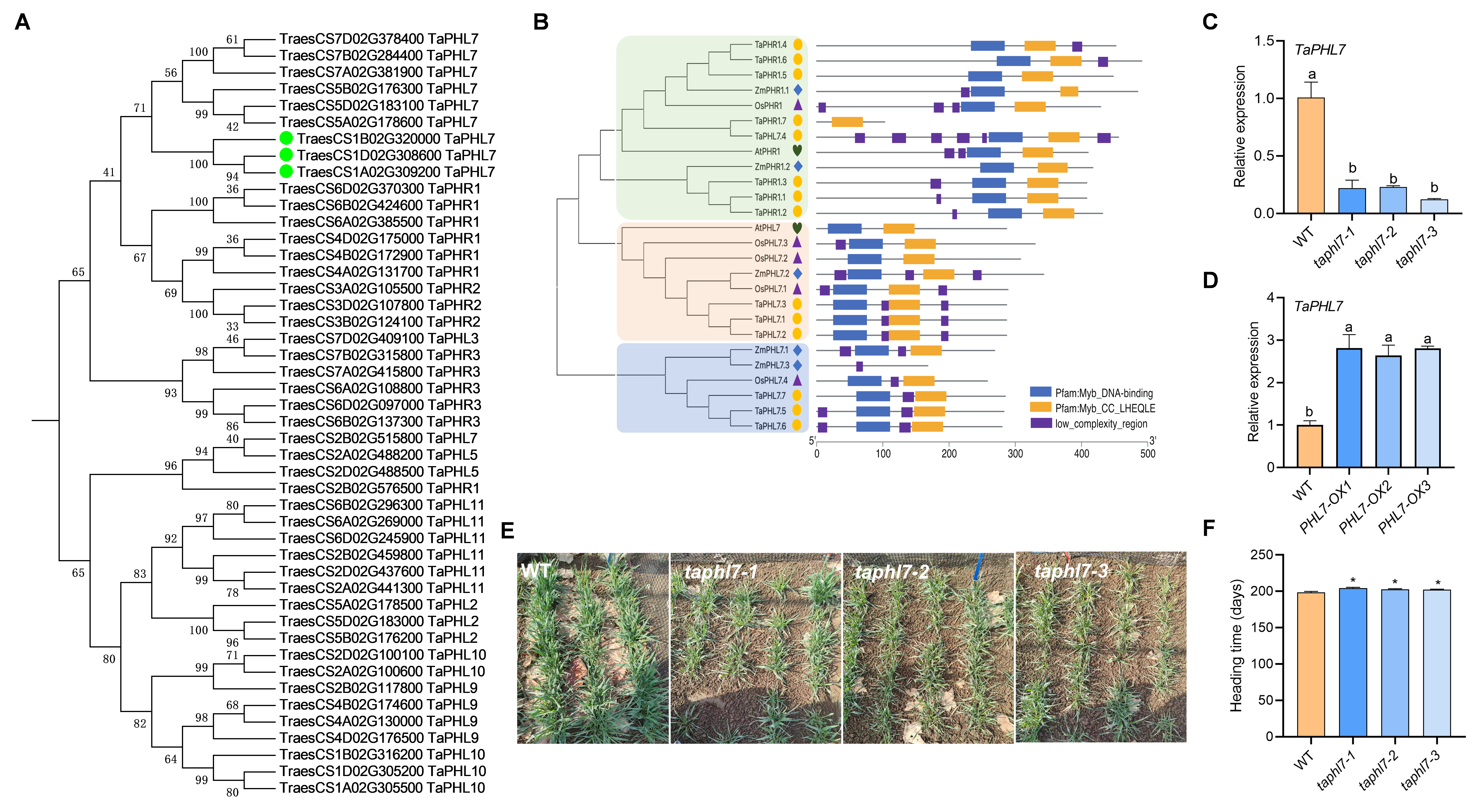


**Supplementary Fig. 2 Phylogenetic analysis of TaPHR and TaPHL proteins. Related to Figure 2.**

**(A)** Phylogenetic analysis of 6 TaPHR and 11 TaPHR-like (TaPHL) proteins in wheat. Green dots indicate the 3 TaPHL7 paralogs. The phylogenetic tree was constructed using MEGA 6.0 software by Neighbor-Jointing method with 1,000 bootstrapping trials.

**(B)** Structural shemes of the conserved domains of PHR and PHL orthologs in Arabidopsis, rice, wheat, and maize. The MYB and CC domains are indicated by blue and orange boxes, respectively.

**(C-D)** Relative expression levels of *TaPHL7* in leaves of wild-type ZM7698 (WT), *taphl7* mutants (C) and *TaPHL7*-overexpressing (*PHL7-OX*) seedlings (D) detected by RT-qPCR.

**(E)** phenotype of the indicated genotypes during the vegetative growth stage.

**(F)** Statistical analysis of heading time at the heading stage.

Data are means with s.d. obtained from two biological replicates, *, *P* < 0.05 (Student’s *t*-test), and different letters indicate *P* ≤ 0.05 (LSD multiple range tests).


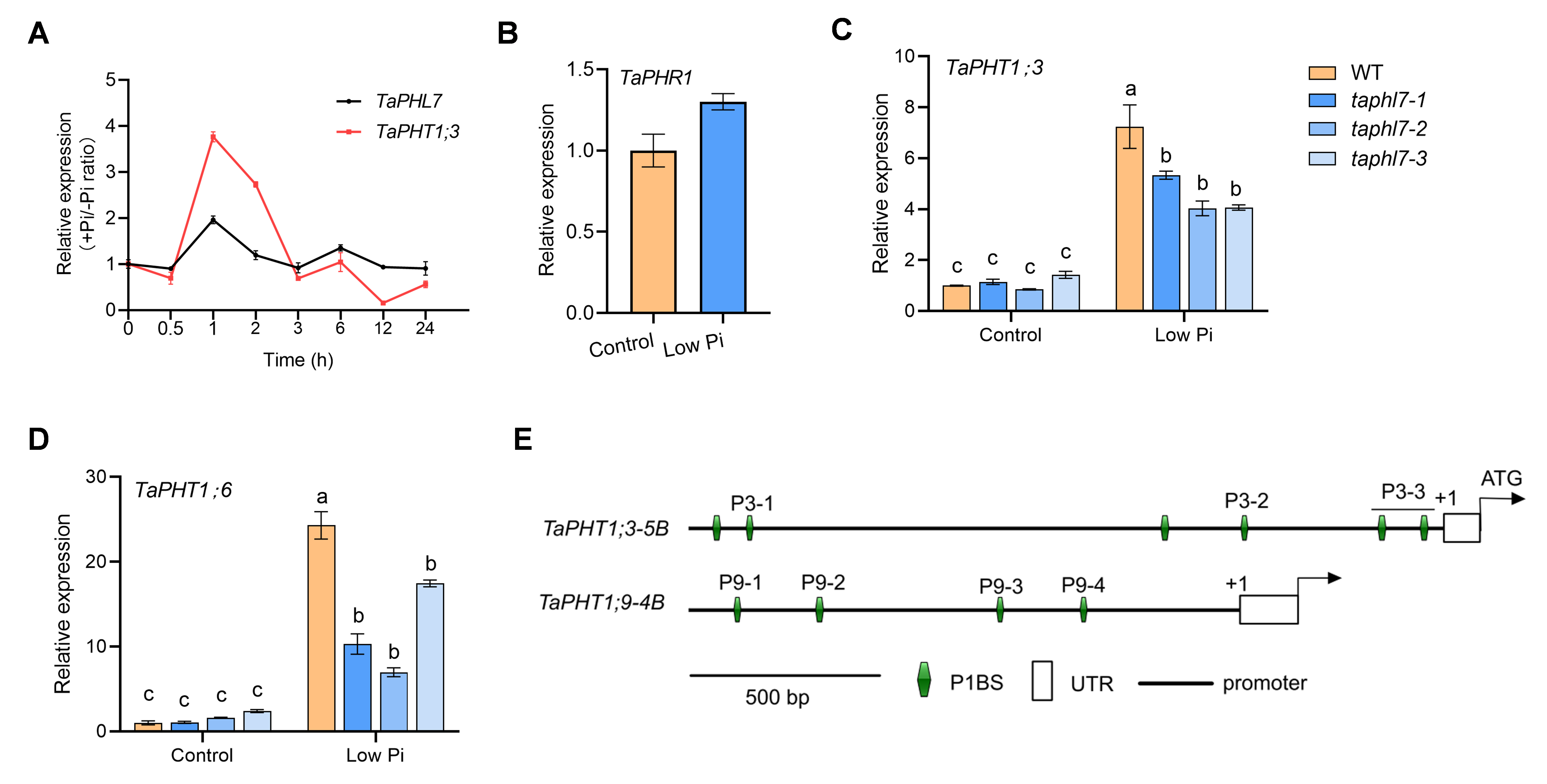


**Supplementary Fig. 3 *TaPHL7* regulates Pi signaling and Pi acquisition. Related to Figure 3.**

**(A)** Relative expression of *TaPHT1;3* and *TaPHL7-1A* in response to phosphate in roots of wild-type ZM7698 seedlings. Sixteen-day-old seedlings grown under phosphate-free condition were transferred to a solution containing 0.20 mM KH_2_PO_4_ and then cultured for the indicated times.

**(B)** Relative expression of *TaPHR1* in roots of 18-day-old wild-type ZM7698 seedlings grown under Control (0.20 mM KH_2_PO_4_) or Low Pi (0.01 mM KH_2_PO_4_) conditions. Data are means with s.d. obtained from two biological replicates, and each replicate consists of at least fifteen plants.

**(C-D)** Relative expression levels of *TaPHT1;3* (C) and *TaPHT1;6* (D) in roots of wild-type ZM7698 (WT) and *taphl7* mutants grown under Control (0.20 mM KH_2_PO_4_) or Low Pi (0.01 mM KH_2_PO_4_) conditions. Seeds were imbibed for 1 day and grown in deionized water for 5 days, followed by cultured under Control or Low Pi conditions for 12 days. Data are means with s.d. (*n* = 3 replicates). Different letters indicate *P* ≤ 0.05 (LSD multiple range tests).

**(E)** Structural scheme of the promoters of *TaPHT1;3-5B* and *TaPHT1;9-4B* genes. The P1BS elements for TaPHL7 binding in the promoter are indicated by green bars. Open boxes indicate the 5’-untranslated regions.


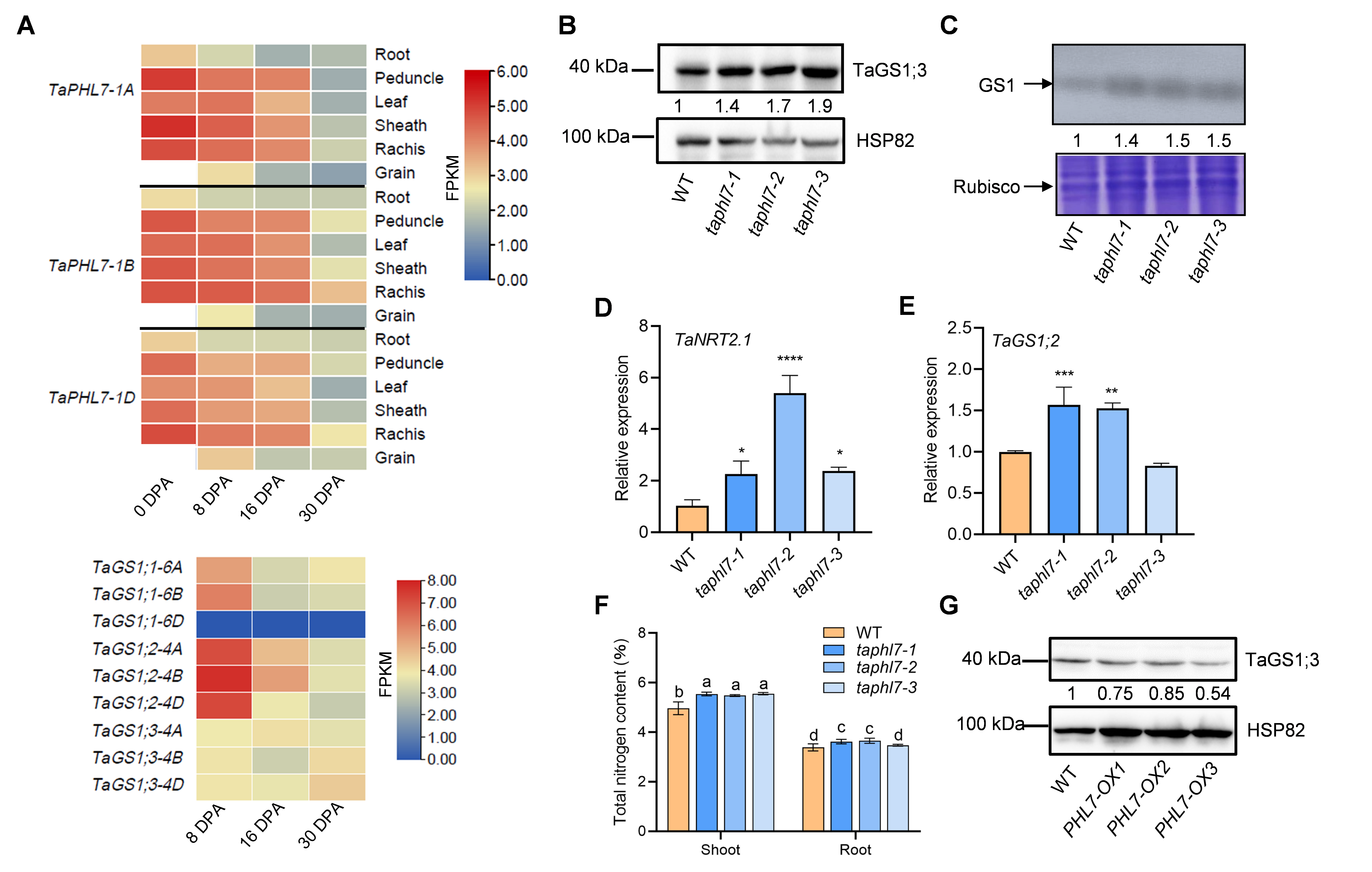


**Supplementary Fig. 4 *TaPHL7* regulates nitrogen metabolism. Related to Figure 4.**

**(A)** Transcriptional profile of three *TaPHL7* homologous genes in the indicated tissues and organs (top) and three *TaGS1* homologous genes (bottom) in developing grain collected at 0-, 8-, 16-, or 30-day post anthesis (DPA).

**(B)** Immunoblotting analysis of TaGS1;3 protein in developing grain of wild-type ZM7698 (WT) and *taphl7* plants at 16-day post anthesis. HSP82 is used as a loading control. The relative levels of TaGS1;3 protein are shown below the blot.

**(C)** Analysis of total GS1 activity in developing grain of WT and *taphl7* plants at 16-day post anthesis. The relative GS activities are shown below the blot.

**(D-E)** Relative expression of *TaNRT2.1* (D) and *TaGS1;2* (E) in roots of WT and *taphl7* seedlings detected by RT-qPCR. Seeds was imbibed for 1 day and grown in deionized water for 5 days, followed by cultured in a hydroponic solution containing sufficient Pi and nitrogen (0.20 mM KH_2_PO_4_ and 2.00 mM Ca(NO_3_)_2_) for 12 days.

**(F)** Analysis of total nitrogen content in shoots and roots of 3-week-old seedlings with the indicated genotypes.

**(G)** Immunoblotting analysis of TaGS1;3 levels in developing grain of WT and *TaPHL7*-overexpressing (*PHL7-OX*) plants at 16-day post anthesis. HSP82 is used as a loading control. The relative levels of TaGS1;3 protein are shown below the blot.

Data in (D-F) are means with s.d., obtained from two biological replicates in (D-E), and 3 biological replicates in F, and each replicate consists of 6 seedlings. *, **, *** *P* < 0.05, *P* < 0.01, *P* < 0.001 (Student’s *t*-test), respectively. Different letters indicate *P* ≤ 0.05 (LSD multiple range tests).


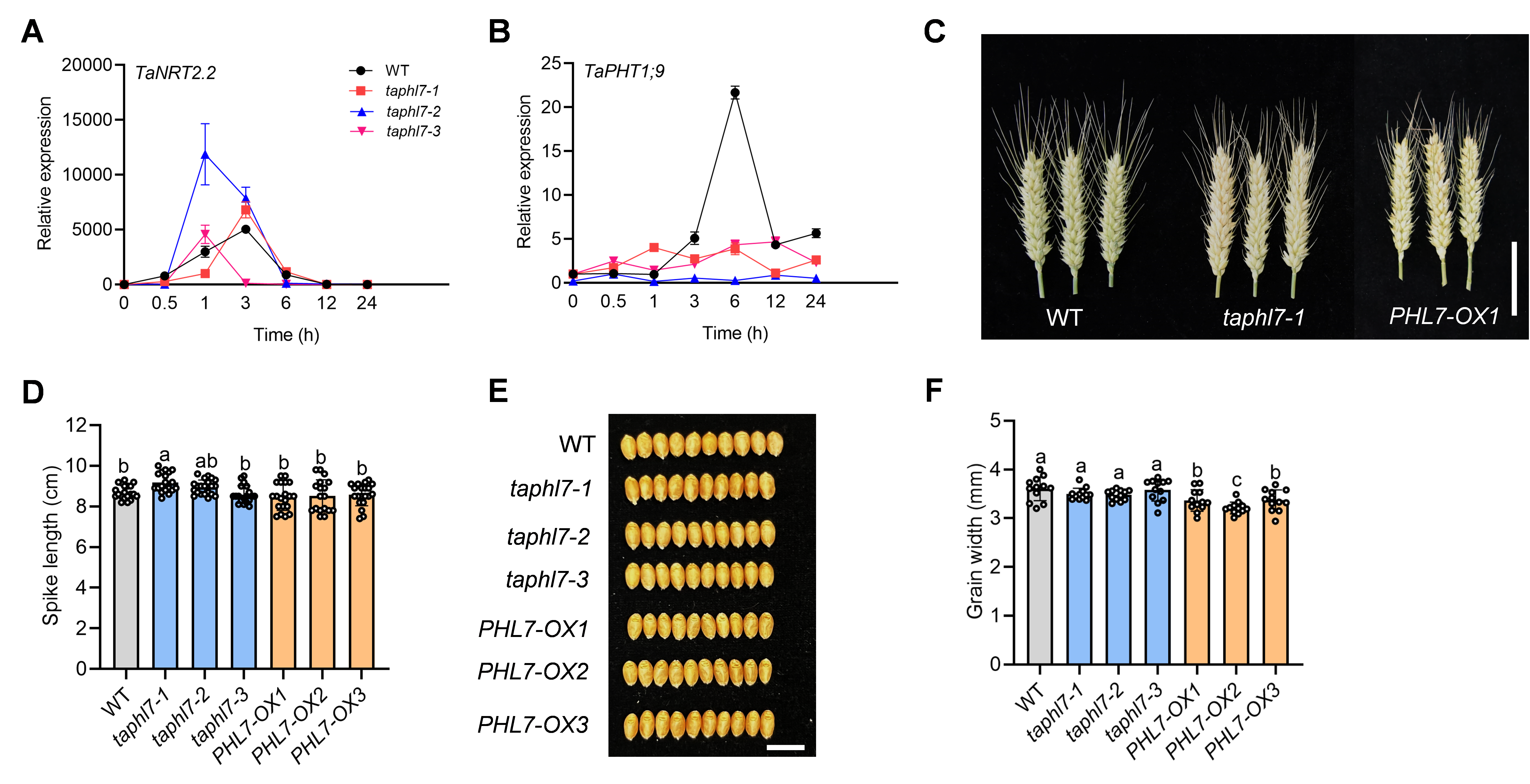


**Supplementary Fig. 5** ***TaPHL7* control maturity and grain yield. Related to Figure 5.**

**(A-B)** Relative expression levels of *TaNRT2.2* (A) and *TaPHT1;9* (B) in roots of wild-type ZM7698 (WT) and *taphl7* seedlings. Sixteen-day-old seedlings grown under nitrogen-free condition were transferred to a solution containing 2.00 mM Ca(NO_3_)_2_ and then cultured for the indicated times.

**(C)** Spikes of WT, *taphl7-1*, and *TaHPL7*-ovexpressing (*PHL7-OX1*) plants at mature stage. Scale bar, 5 cm.

**(D)** Quantitative analysis of spike length of the indicated genotypes.

**(E)** Mature seeds of the indicated genotypes. Scale bar, 1 cm.

**(F)** Statistical analysis of grain width of seeds shown in (E).

All data presented are means with s.d., obtained from 3 to 4 technical replicates in (A-B), and at least 12 plants in (C-F). Different letters indicate *P* ≤ 0.05 (LSD multiple range tests).

**
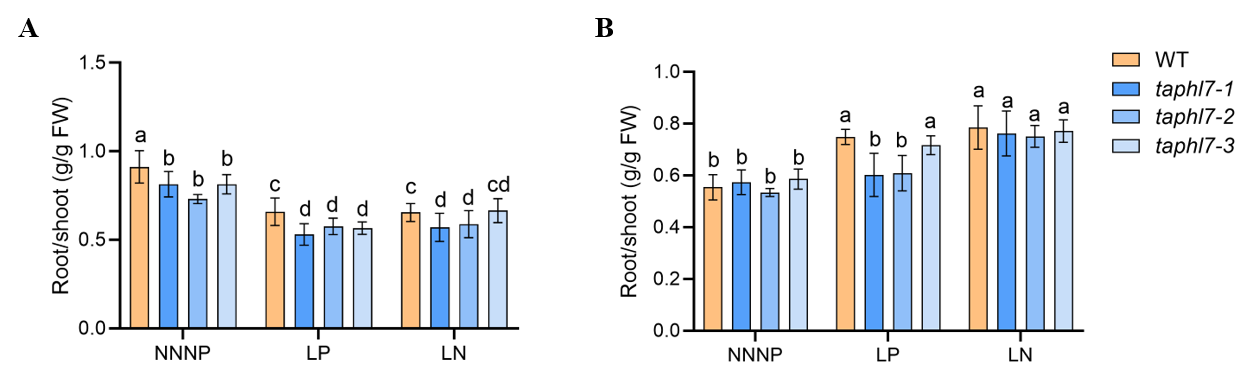
**

**Supplementary Fig. 6 The effect of *TaPHL7* loss-of-function on wheat growth and development under nitrogen and phosphorus treatment conditions. Related to Figure 5.**

**(A-B)** Quantitative analysis of biomass (A) and root/shoot (B) of seedlings. Eighteen-day-old wild-type ZM7698 (WT) and *taphl7* seedlings grown under N-P sufficient (NNNP; 2.00 mM Ca(NO_3_)_2_ and 0.2 mM KH_2_PO_4_), Pi-deficient (LP; 2.00 mM Ca(NO_3_)_2_ and 0.01 mM KH_2_PO_4_), N-deficient (LN; 0.20 mM Ca(NO_3_)_2_ and 0.2 mM KH_2_PO_4_) conditions.


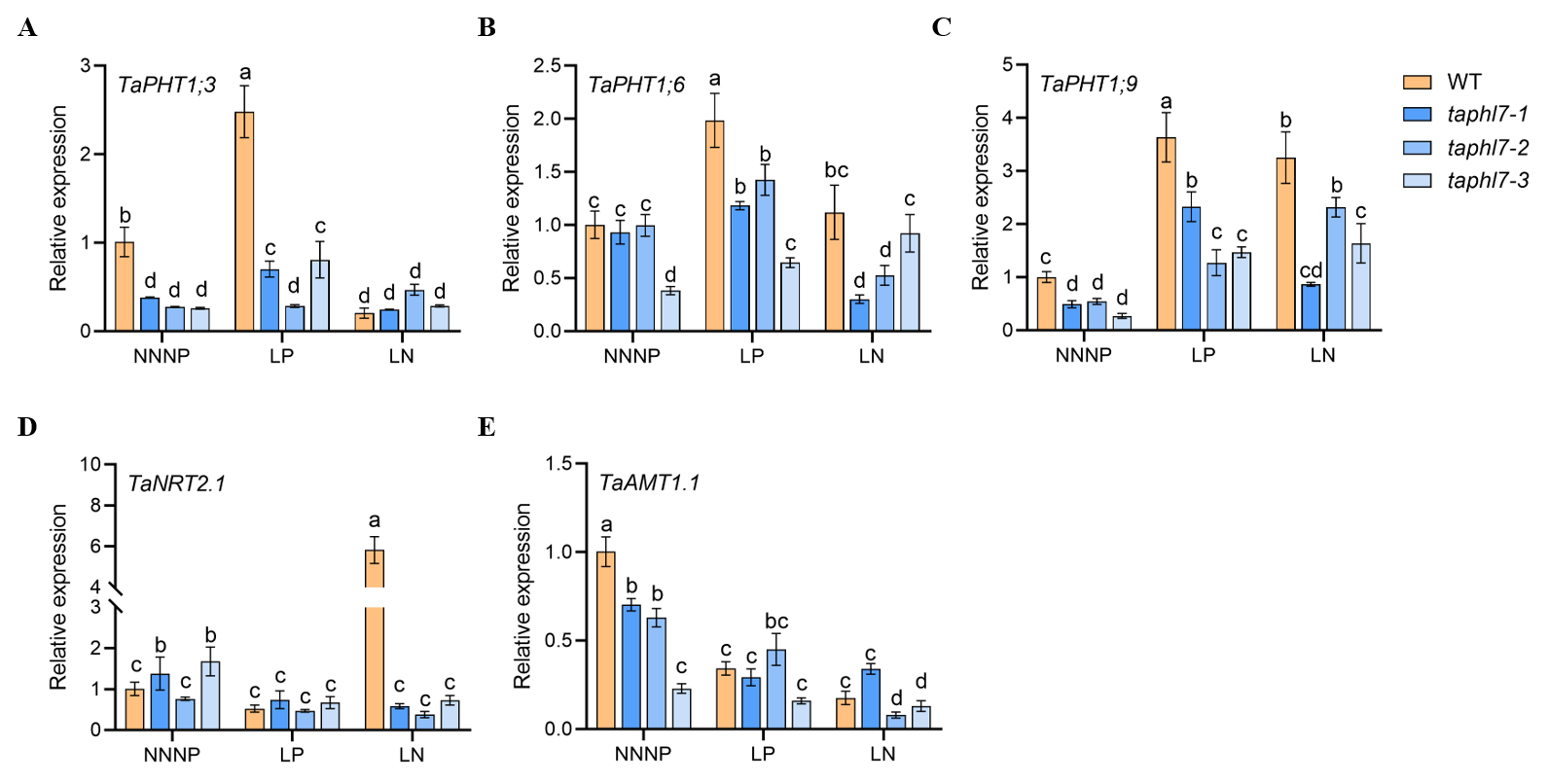


**Supplementary Fig. 7** **Regulation of nitrogen and phosphorus marker genes by TaPHL7 under nitrogen and phosphorus treatment conditions. Related to Figure 5.**

**(A-E)** Relative expression levels of *TaPHT1;3* (A), *TaPHT1;6* (B), *TaPHT1;9* (C)*, TaNRT2.1* (D)*, TaAMT1.1* (E) in roots of 18-day-old WT and *taphl7* mutants grown under Control (NNNP), Low Pi condition (LP) or Low N condition (LN).

**
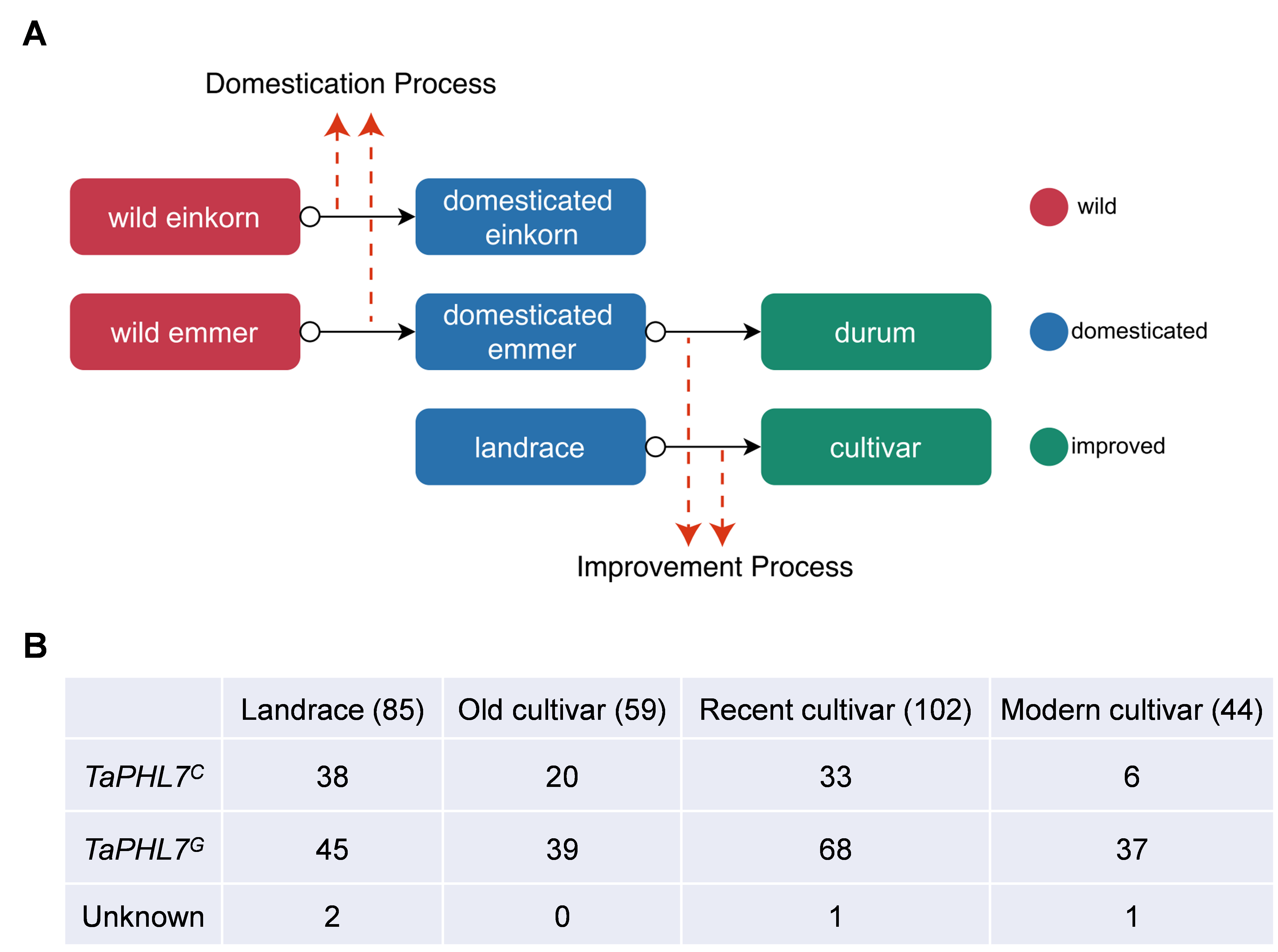
**

**Supplementary Fig. 8 Evolution of *TaPHL7* during wheat domestication and improvement. Related to Figure 6.**

**(A)** A simplified route of domestication and improvement processes of the *Triticum* genus.

**(B)** Distribution of two *TaPHL7* haplotypes (*TaPHL7^C^* and *TaPHL7^G^*) in four wheat historical populations. Number of germplasms with missing genomic sequence of *TaPHL7* are listed at the bottom.

**Supplementary Table 1 Candidate P1BS elements in the promoters of selected genes**


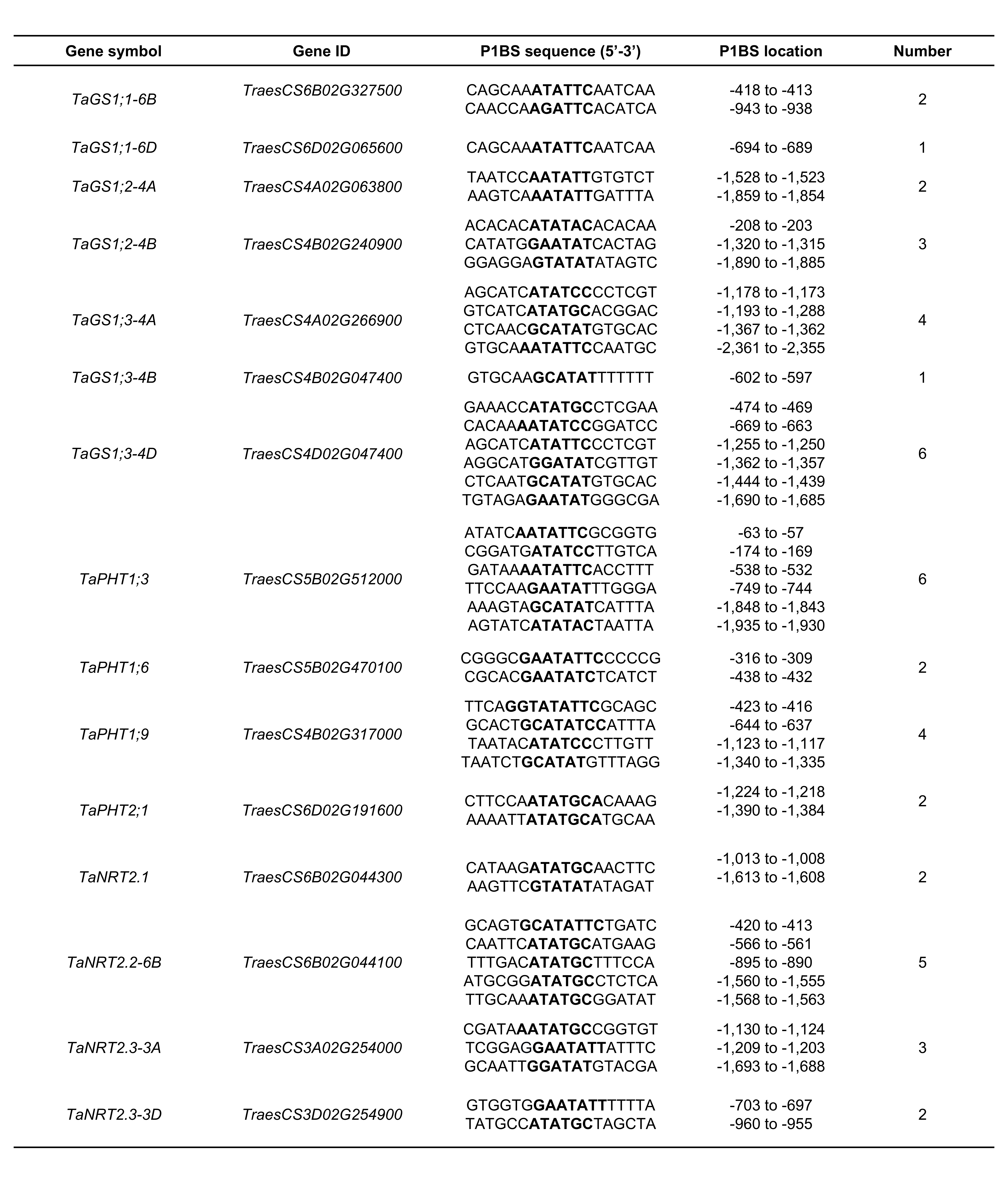


**Supplementary Table 2 Primers used in this study.**

| **Primer name** | **Sequences (5'-3')** |
| --- | --- |
| **Primers used for generation and identification of *taphl7* mutants and *PHL7-OX* plants** | |
| PHL7-gRNA1-F | TGCATGAGCCCTCTGTGGTGCAAA |
| PHL7-gRNA1-R | AAACTTTGCACCACAGAGGGCTCA |
| PHL7-gRNA2-F | TGCAGTGGATGCAATCGCCCAACT |
| PHL7-gRNA2-R | AAACAGTTGGGCGATTGCATCCAC |
| TaU3- F | CTATGTTACTAGATCAAGCTTGAATTCATCCTCACGTTCAACACC |
| TaU3- R | CTGCAGGCATGCAAGCTTCCATCCACTCCAAGCTCTTGAAA |
| PHL7-1A-F | ACCACCAAATTGCAGGTTCT |
| PHL7-1A-R | TTCTTGGCCTTGCTCATGTGTAT |
| PHL7-1B-F | CGACATACAACCAGCAAGGTG |
| PHL7-1B-R | ATGCTCAGTGCGCTGTATTTA |
| PHL7-1D-F | TACAACCACCAAAGTGCAGGT |
| PHL7-1D-R | TGCTGTATTTACCTTTTGCCGTC |
| PHL7-F | ggagtgagtacggtgtgcGTTGCAGTACTCTCATGATG |
| PHL7-R | GAGTTGGATGCTGGATGGCTGACAAGGTTCATGAGAAACC |
| PHL7-OX-F | GATCCCCGGGTACCGAGCTCATGATGTACCATGCTAAGAAG |
| PHL7-OX-R | CGATCGGGGAAATTCGAGCTCTTATGACTTGCTCAAACCAG |
| PHL7-CDS-F | ATGATGTACCATGCTAAGAAGTTTTCTG |
| PHL7-CDS-R | TTATGACTTGCTCAAACCAGGGT |
| **Primers used for RT-qPCR** | |
| Actin-F | GAACCTCCACTGAGAACAACATTACC |
| Actin-R | GTTCCAATATATGAGGGATACACGC |
| TaTEF-F | GGTTGTGGAGACCTTTGCTACTTAC |
| TaTEF-R | AACAGCCACAGTTTGCCTCAT |
| TaSPX3-F | GTGGAAGGACGAGTTCCTGAGC |
| TaSPX3-R | TCCCGGTGTGTGATGATGAAGAA |
| TaPHT1.3-F | CTGCTCACCTACTACTGGCGG |
| TaPHT1.3-R | CCCGAAGTCGTTGGCTCC |
| TaPHT1.6-F | TACTACTGGCGGATGAAGATGC |
| TaPHT1.6-R | GTTGCGGTCGTCGGCG |
| TaPHT1;9-F | CAGAAGAAGCCCGAGACCG |
| TaPHT1;9-R | TGTACACCAGCCTAAGCTTCGA |
| TaNRT2.2-F | CCTTGGTATCATCTCCGGGC |
| TaNRT2.2 -R | GAGGGTGACAGGAAGAGTGC |
| TaNRT2.1-F | CGCTGTCTTTGGTGTTGC |
| TaNRT2.1 -R | GACCCTTGCCCTTCTCCT |
| TaGS1.2-F | GACAACTTCCTTGTTATGTGCCAC |
| TaGS1.2-R | TGTGCCTCTTGTTCGTGGG |
| TaGS1;3-F | CTGTGACTGCTATGCGCCTAAC |
| TaGS1;3-R | CCGCGTTGTACCGCTTGT |
| TaPHL7-A1-F | CTCTTCCGTGCCGTACTCTC |
| TaPHL7-A1-R | GGATCCACCGAAAGCTCCAA |
| TaPHR1-F | GGATGGTCTATCCCAAAGTATC |
| TaPHR1-R | GCTGCCTTAAGCACAGATCC |
| **Primers used for ChIP-qPCR** | |
| PUC-TaPHL7-GFP-F | CGCCACTAGTGGATCCATGATGTACCATGCTAAGAAGTTTTCTG |
| PUC-TaPHL7-GFP-R | TACATCCCGGGAGCGGTACCTGACTTGCTCAAACCAGGGT |
| PHT1.3pro-1F | ATGCTACAACTACGGAGGGC |
| PHT1.3pro-1R | CCGAGGCTGTAAAACTCCGA |
| PHT1.3pro-2F | CGCGTCTCCAAAATTCATTTG |
| PHT1.3pro-2R | GCAGCGGGTACCTACAAGAA |
| PHT1.3pro-3F | AAGTGCAGCATTTTGACGCA |
| PHT1.3pro-3R | GGCCGGCTGTCTGGTATAAG |
| PHT1;9pro-1F | TGCCATGTAGGTACGGAGGA |
| PHT1;9pro-1R | TGCCTTGACAGCAACAGCTA |
| PHT1;9pro-2F | TGTCTGGAGGACCTAGCACT |
| PHT1;9pro-2R | TGGCAGGAAGAAGGTAAGCG |
| PHT1.9pro-3F | TGTGACAACCATTTCATGCACC |
| PHT1.9pro-3R | TGCTTGGTTCTAGCACAGGA |
| PHT1.9pro-4F | TAGTACGGAGCACGGCTTTC |
| PHT1.9pro-4R | AGGTGCATGAAATGGTTGTCAC |
| GS1.3-4Dpro-P1-F | ACCCAGGCATTTCAAAGAAATAGTG |
| GS1.3-4Dpro-P1-R | CAACTACCGAAGACACCGCAC |
| GS1.3-4Dpro-P2-F | AGATGTACTCGTCCCACAAATC |
| GS1.3-4Dpro-P2-R | AGTTGAACTGTGTTCAACTGCC |
| GS1.3-4Dpro-P3-F | TCCCTTGATCGATGCCTTGAA |
| GS1.3-4Dpro-P3-R | GGACGAATTTGAGGATGAAAAATCG |
| GS1.3-4Dpro-P4-F | GAATAGCTCGTCGGGGACAAA |
| GS1.3-4Dpro-P4-R | GACATCGAGTGTTGAATCGGC |
| GS1.3-4Dpro-P5-F | CATGTATGCTTCCGGTTTATGGG |
| GS1.3-4Dpro-P5-R | CGTGATGATATGGACAGCCAAC |
| GS1.3-4Dpro-P6-F | GAGGAATGACACGACAGGCA |
| GS1.3-4Dpro-P6-R | GTTCTCTTGTCCCGGCGTC |
| GS1.3-4Dpro-P0-F | GAGGGGGAACAACATCCTGG |
| GS1.3-4Dpro-P0-R | TACCGCTTGTTGCTCGGAAT |
| **Primers used for Dual-Luciferase Reporter assay** | |
| LUC-GS1.3-4D-F | CTATAGGGCGAATTGGGTACCCTGTTCTCACCCCTTGGA |
| LUC-GS1.3-4D-R | ATCTCCACCGCGGTGGCGGCCGCCGAGTCCGTGATGATATG |
| LUC-GS1.3-4D-F(Δ4) | CAAAATAGTCGTCCATCAGCATCCCTCGTGCCCTATGCCGATTCAACACTCGATG |
| LUC-GS1.3-4D-R(Δ4) | CATCGAGTGTTGAATCGGCATAGGGCACGAGGGATGCTGATGGACGACTATTTTG |
| LUC-GS1.3-4D-F(Δ5) | TATGACATTGAATGACACAAAGGATCCTAGACTTTTGGCGACGTTTTGATGATGAA |
| LUC-GS1.3-4D-R(Δ5) | TTCATCATCAAAACGTCGCCAAAAGTCTAGGATCCTTTGTGTCATTCAATGTCATA |
| LUC-PHT1;3-5B-F | TTCCTGCAGCCCGGGGGATCCAGAAATGTCACAAATTCCAAG |
| LUC-PHT1;3-5B-R | CGCTCTAGAACTAGTGGATCCAGATGCAGAAGAGGTCGTAG |
| LUC-PHT1;9-4B-F | TTCCTGCAGCCCGGGGGATCCAGGTTCTGCTCTCACGATGATA |
| LUC-PHT1;9-4B-R | CGCTCTAGAACTAGTGGATCCACGTTGAGCTGTTCAGTCGC |
| Flag-PHL7-F | ATACACCAAATCGACTCTAGAATGATGTACCATGCTAAGAAGTTTTCTG |
| Flag-PHL7-R | GTCTTTGTAATCCATGGTACCTTATGACTTGCTCAAACCAGGGT |
| **Primers used for Y1H** | |
| pGADT7-PHL7-F | GTACCAGATTACGCTCATATGATGATGTACCATGCTAAGAAGTTTTCTG |
| pGADT7-PHL7-R | ATGCCCACCCGGGTGGAATTCTTATGACTTGCTCAAACCAGGGT |
| pAbAi-GS1;3-4D-F | AGCACATGCCTCGAGGTCGACTTGACTTAGCAGACGCGACATC |
| pAbAi-GS1;3-4D-R | GAAAAGCTTGAATTCGAGCTCCATGGCTACCTCTTCTTCTTCTTCC |
| **Primers used for Subcellular Localization analysis** | |
| 163-PHL7-GFP-F | GTGTTACTTCTGCAAAGCTTATGATGTACCATGCTAAGAAGTTTTCTG |
| 163-PHL7-GFP-R | CCCTTGCTCACCATGGATCCTGACTTGCTCAAACCAGGGT |

**Supplementary Table 3 List of *TaGS1;3*-interacting factors screened by Y1H**

| Positive Number | Protein name | Molecular function |
| --- | --- | --- |
| 1 | Probable 4-hydroxy-tetrahydrodipicolinate reductase 1, chloroplastic | Catalyzes the conversion of 4-hydroxy-tetrahydrodipicolinate (HTPA) to tetrahydrodipicolinate |
| 1 | Villin-2 | Actin filament binding |
| 1 | GSNAP | Gamma-soluble NSF attachment protein |
| 1 | Thioredoxin H2-2 | Probable thiol-disulfide oxidoreductase that may be involved in the redox regulation of a number of cytosolic enzymes. |
| 1 | Uncharacterized protein (TRITD_6Bv1G181070) | Transmembrane transporter activity |
| 2 | E3 ubiquitin-protein ligase UPL3 | HECT domain-containing protein |
| 1 | Probable cellulose synthase A catalytic subunit 5 [UDP-forming] | Catalytic activity |
| 1 | Uncharacterized protein | Protein phosphatase inhibitor activity |
| 1 | V-type proton ATPase subunit G1(VHA-G1) | Proton-exporting ATPase activity, phosphorylative mechanism |
| 1 | Vacuolar sorting protein 18 (VPS18) | Protein-macromolecule adaptor activity, metal ion binding |
| 1 | DNA-directed RNA polymerase subunit beta (rpoC2) | DNA-dependent RNA polymerase catalyzes the transcription of DNA into RNA using the four ribonucleoside triphosphates as substrates |
| 2 | 26S proteasome regulatory subunit 6A homolog (TBP1) | ATPase activity, ATP binding |
| 1 | Transmembrane 9 superfamily member | Not annotated code |
| 2 | Probable manganese-transporting ATPase PDR2 (PDR2) | Mediates manganese transport into the endoplasmic reticulum |
| 2 | **Myb family transcription factor PHL7** | DNA-binding transcription factor activity |
| 1 | Cyclic nucleotide-gated ion channel 1(CNGC1) | Acts as a cyclic nucleotide-gated ion channel |
| 2 | Nuclear pore complex protein NUP93A(NUP93A) | Structural constituent of nuclear pore |
| 1 | 3-ketoacyl-CoA thiolase 2, peroxisomal | Acetyl-CoA C-acyltransferase activity |
| 1 | Endoplasmic reticulum oxidoreductin-1(AERO1) | Essential oxidoreductase that oxidizes proteins in the endoplasmic reticulum to produce disulfide bonds |
| 1 | F-box protein At3g58530 | SCF-dependent proteasomal ubiquitin-dependent protein catabolic process |
| 1 | PITH domain-containing protein | Not annotated code |
| 1 | Glyceraldehyde-3-phosphate dehydrogenase | Protein localization to the membrane |
| 1 | Chlorophyll a-b binding protein (TRITD_7Bv1G056370) | The light-harvesting complex (LHC) functions as a light receptor, capturing and delivering excitation energy to photosystems with which it is closely associated |
| 1 | COX5C | Cytochrome c oxidase subunit 5C |
| 1 | CBP1, Serine carboxypeptidase 1 | Related to the degradation of small peptides (2-5 residues) or storage proteins in the embryo |
| 1 | molybdate-anion transporter | chlorophyll binding |
| 1 | Putative MO25-like protein At5g47540 | Protein serine/threonine kinase activator activity |
| 1 | Puroindoline-B | Serine-type endopeptidase inhibitor activity |
| 1 | F-box/kelch-repeat protein OR23 | Protein degradation; cell cycle regulation: signal transduction |
| 1 | Uncharacterized protein | Sequence-specific DNA binding, transcription regulatory region sequence-specific DNA binding |
| 2 | BHLH domain-containing protein | DNA-binding transcription factor activity |
| 1 | PDZ6 domain-containing protein | Serine-type endopeptidase activity |
